# Virtual Standardized Patients Versus Traditional Academic Training for Improving Clinical Competence Among Traditional Chinese Medicine Students: Prospective Randomized Controlled Trial

**DOI:** 10.2196/43763

**Published:** 2023-09-20

**Authors:** Han Yang, Xiang Xiao, Xuanyu Wu, Xiaoxu Fu, Quanyu Du, Yan Luo, Bin Li, Jinhao Zeng, Yi Zhang

**Affiliations:** 1 Hospital of Chengdu University of Traditional Chinese Medicine Chengdu University of Traditional Chinese Medicine Chengdu China; 2 Department of Chinese Internal Medicine Clinical Medical School of Chengdu University of Traditional Chinese Medicine Chengdu University of Traditional Chinese Medicine Chengdu China; 3 Clinical Medical School of Chengdu University of Traditional Chinese Medicine Chengdu University of Traditional Chinese Medicine Chengdu China; 4 Clinical Skill Center Clinical Medical School of Chengdu University of Traditional Chinese Medicine Chengdu University of Traditional Chinese Medicine Chengdu China

**Keywords:** virtual standardized patients, clinical competence, traditional Chinese medicine, medical education

## Abstract

**Background:**

The practical training course of internal medicine of traditional Chinese medicine (PTC-IMTCM) is primarily based on traditional case teaching, which can be stressful for teachers. The use of virtual standardized patient (VSP) applications could be an alternative; however, there is limited evidence regarding their feasibility and effectiveness.

**Objective:**

This study aimed to build a VSP-TCM application according to the characteristics of PTC-IMTCM and the needs of students and to compare its efficacy with that of traditional teaching in improving TCM clinical competence among students.

**Methods:**

A prequestionnaire investigation was conducted before the course, and a VSP-TCM system was developed based on the results of the questionnaire. A randomized controlled trial was then conducted between February 26, 2020, and August 20, 2021. A total of 84 medical students were included and were divided into 2 groups: an observation group, trained with VSP-TCM (n=42, 50%), and a control group, trained with traditional academic training (n=42, 50%). Formative and summative assessments were conducted to evaluate teaching effectiveness. After completing the course, the students were administered a questionnaire to self-assess their satisfaction with the course. A questionnaire was also administered to 15 teachers to uncover their perspectives on VSP-TCM.

**Results:**

All participants completed the study. In the formative assessment, the VSP-TCM group performed better in medical interviewing ability (mean 7.19, SD 0.63, vs mean 6.83, SD 0.81; *P*=.04), clinical judgment (mean 6.48, SD 0.98, vs mean 5.86, SD 1.04; *P*=.006), and comprehensive ability (mean 6.71, SD 0.59, vs mean 6.40, SD 0.58; *P*=.02) than the control group. Similarly, in the summative evaluation, the VSP-TCM group performed better in the online systematic knowledge test (OSKT; mean 86.62, SD 2.71, vs mean 85.38, SD 2.62; *P*=.046), application of TCM technology (mean 87.86, SD 3.04, vs mean 86.19, SD 3.08; *P*=.02), TCM syndrome differentiation and therapeutic regimen (mean 90.93, SD 2.42, vs mean 89.60, SD 2.86; *P*=.03), and communication skills (mean 90.67, SD 4.52, vs mean 88.24, SD 4.56; *P*=.02) than the control group. There was no significant difference in medical writing between both groups (mean 75.07, SD 3.61, vs mean 75.71, SD 2.86; *P*=.37). The postcourse feedback questionnaire indicated that VSP-TCM can better enhance students’ TCM thinking ability (n=39, 93%, vs n=37, 88%; *P*=.002), medical history collection (n=38, 90%, vs n=30, 72; *P*=.001), syndrome differentiation and treatment and critical thinking (n=38, 90%, vs n=37, 88%; *P*=.046), comprehensive clinical application ability (n=40, 95%, vs n=36, 86%; *P*=.009), interpersonal communication skills (n=36, 86%, vs n=28, 67%; *P*=.01), and autonomous learning ability (n=37, 88%, vs n=28, 67%; *P*=.01) than traditional academic training. Similarly, the teachers held a positive perspective on VSP-TCM.

**Conclusions:**

VSP-TCM enhances students’ TCM clinical competence and dialectical thinking and improves their ability to work autonomously. Moreover, the VSP-TCM system is feasible, practical, and cost-effective and thus merits further promotion in TCM education.

## Introduction

Clinical practice is the most crucial experience for undergraduate medical students [1]. However, some challenges, such as students’ negative emotions when interacting with real patients, affect teaching effects [2]. Therefore, trained actors are incorporated as standardized patients (SPs) to not only train students for medical education but also help reduce their anxiety, recreate the medical environment, and improve teaching efficiency by providing real-time feedback on students’ diagnoses and therapeutic activities [3,4]. However, SP applications have some limitations. First, SP requires exceptional role-playing talent, which makes recruiting SP challenging [5]. Second, developing a professional SP takes time, money, and effort [6]. Third is the lack of SP standardization [7]. In our previous teaching practices where we implemented SP, the results were encouraging [8,9]; however, the aforementioned limitations remain a concern.

An alternative training approach could be the use of virtual standardized patient (VSP) programs, which use computerized characters for SP encounters [3]. Compared to SPs, a VSP program has significant advantages as it requires fewer personnel and resources, is available at any time, and is highly customizable [10]. In addition, it can offer highly interactive and engaging experiences to trainees [3]. A VSP program is significantly useful in clinical scenarios where SPs are difficult to use; it facilitates effective communication between doctors and patients with rare diseases, speech disorders, and mental disorders [11]. Furthermore, compared to SPs, a VSP program is more standardized because educators control its design, programming, delivery, and use [12]. Reger et al [3] used a VSP program to help health care workers improve their motivational interviewing skills. Guetterman et al [13] proposed that VSP programs can improve medical students’ empathic communication abilities. Du et al [14] assessed the history-taking skills of nursing students in China using a VSP program and found that the VSP program was effective in achieving relatively objective, standardized, and consistent education evaluation [14].

Traditional Chinese medicine (TCM) is a comprehensive medical system that emphasizes the holistic concept of harmony between humans and nature. Its core principle is to maintain the balance between yin and yang, promote the circulation of qi and blood, and coordinate the different functions of various organs of the body. TCM clinical skills, such as inspection, auscultation and olfaction, palpation, and inquiry, are usually used to assess an individual’s overall health status. TCM practitioners often adopt various therapeutic methods to treat patients, such as herbal medicine, acupuncture, moxibustion, and *tuina* [15,16]. The role of SPs in TCM education is a frontier topic in the Chinese medical education system [17]. In 2012, Liu et al [18] took the lead in establishing occupational standardized patients (OSPs) for TCM education, following which the first batch of TCM surgical students trained with OSPs was cultivated. Chen et al [19] used OSPs to assess the educational effect of objective structured clinical examination on TCM clinical training. Since then, we have established a group of OSPs suitable for the practical training course of internal medicine of traditional Chinese medicine (PTC-IMTCM), which has significantly improved students’ medical records and their ability to treat based on syndrome differentiation [8].

However, using SPs in TCM education has several limitations; therefore, VSPs present a viable alternative. In the past, VSPs have not been used in TCM education due to technical limitations and the lack of promotion and traditional view. With the implementation of education informatization, an education reform, VSP programs have become more mature and widely acknowledged [20]. Simultaneously, with the development of medical case databases, a VSP program is now capable of offering users a wider range of clinical scenarios. In addition, a VSP program overcomes time and space constraints, allowing students to engage in clinical exercises at their convenience. Our team also used OSPs and student standardized patients (SSPs) in early-stage teaching, which yielded positive student learning outcomes [8,9]. However, OSPs and SSPs require costly personnel and material resources, significantly limiting their promotion, whereas VSPs are a low-cost tool [21]. The Chengdu University of Traditional Chinese Medicine (CDUTCM) is one of the few TCM universities that launched the VSP-TCM project.

In 2019, we formally established the VSP-TCM system and cultivated the ﬁrst batch of students trained using TCM-VSP. Subsequently, we integrated the VSP-TCM system into the PTC-IMTCM course and conducted training in VSP-TCM mode. In this study, we conducted a multifaceted analysis to compare the effects of VSP-TCM and traditional academic training on students’ clinical competence. We hypothesized that medical students who receive VSP-TCM training would achieve greater progress in clinical competency than those who receive traditional academic training. Our findings are expected to provide a reduced-cost and high-efficiency training mode in TCM medical education.

## Methods

### Study Design

**Figure 1:**
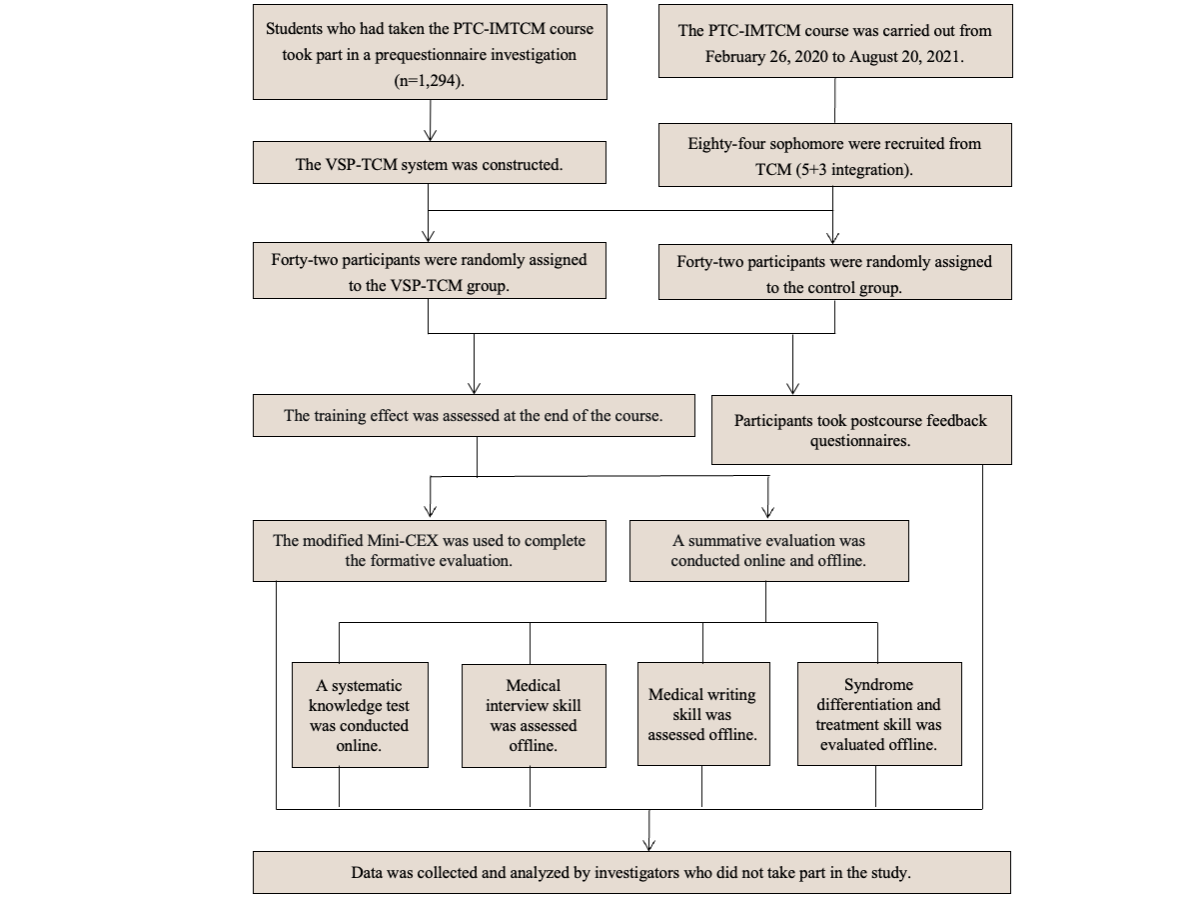
Flow diagram of the study. PTC-IMTCM: practical training course of internal medicine of traditional Chinese medicine; VSP-TCM: virtual standardized patient of traditional Chinese medicine; TCM: traditional Chinese medicine; Mini-CEX: mini-clinical evaluation exercise.

First, we conducted a questionnaire investigation to gain the perspectives of TCM students who have taken the PTC-IMTCM course. The final version of the VSP-TCM system was designed and developed based on the characteristics of TCM and the results of the questionnaire investigation [22]. Subsequently, we conducted a single-blind, 2-group, parallel-training randomized controlled trial to compare the effectiveness of VSP-TCM and traditional academic training in improving clinical competence among TCM medical students. Figure 1 shows the study flow.

### Ethical Considerations

The study was approved by the Ethics Committee of Chengdu University of Traditional Chinese Medicine (CDUTCM; approval no. 25382) and adhered to the principles of the Declaration of Helsinki. Curriculum planning adhered to the guidelines set by the “Undergraduate Medical Education Standards - Traditional Chinese Medicine” issued by the National Advisory Committee on Higher Traditional Chinese Medicine Education of the Ministry of Education [23] and the “Eight-Year Undergraduate Talent Training Guide of Traditional Chinese Medicine” issued by the CDUTCM. The study followed the Consolidated Standards of Reporting Trials of Electronic and Mobile Health Applications and Online Telehealth (CONSORT-EHEALTH) checklist to report its findings [24]. All participants provided written informed consent before participating in the study.

### Development of the VSP-TCM System

#### Prequestionnaire Investigation

A questionnaire was administered to 1628 medical students who had taken the PTC-IMTCM course. The questionnaire was formulated based on the characteristics and teaching objectives of the course, which included basic information, learning methods, learning habits, learning interests, and acceptance and demand for VSP-TCM. Students who had completed the course were chosen as survey subjects over those who did not complete it, because the former were more familiar with the contents and teaching methods of the course. They could also provide better feedback on course improvement. The results of the questionnaire were used to design the detailed functions and modules of the VSP-TCM system.

#### Design of the VSP-TCM System

The teaching goal of VSP-TCM is to enhance the clinical competence of TCM students. We constructed a case database that included diseases that met the requirements of the latest version of the physician qualification examination outlines. The included diseases were all foundational. Subsequently, we developed an intelligent human-computer interaction environment where users could send instructions by text or voice, such as “What is the main reason for your coming to see a doctor this time?” The VSP-TCM system responded to the user’s commands and simulated a clinical diagnosis, including history taking, physical examination, auxiliary examination, diagnosis and differential diagnosis, and treatment. The VSP-TCM system also summarized the systematic knowledge and evaluated the user’s performance in real time.

We collaborated with Shanghai Dream Road Digital Technology Co. Ltd. to complete the development of the VSP-TCM system. Autodesk Maya was used for scene and character modeling, and Adobe Animate CC technology was used for program synthesis. Microsoft Windows 7 or higher was used as the computer operating system, and Microsoft Internet Explorer 8.0 or higher was used as the browser. When using the system, the students wore headsets with microphones (Lenovo).

#### User Flow of the VSP-TCM System

**Figure 2:**
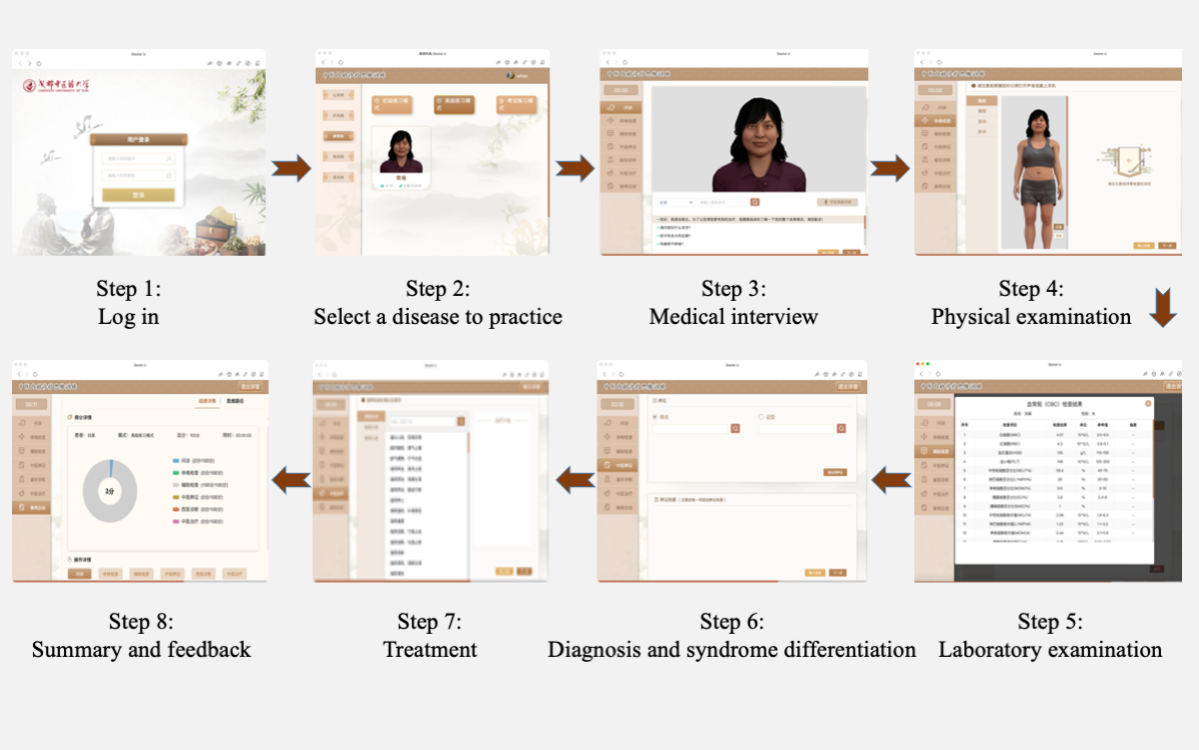
Operation procedures of the VSP-TCM system.

The operation procedures of the VSP-TCM system (Figure 2) were as follows: (1) After logging in, select a disease to practice; (2) send commands via voice or text to obtain medical history, including chief complaints, current medical history, past medical history, personal history, and family history; (3) perform targeted physical examinations according to the collected medical history, including vital signs, various systems, and TCM tongue and pulse; (4) conduct appropriate auxiliary examinations, including laboratory and imaging examinations; (5) perform a diagnosis according to the information collected, including a diagnosis and differential diagnosis based on the theory of TCM and Western medicine (WM) and TCM syndrome differentiation; (6) create treatment plans based on specific diagnoses, including TCM and WM treatment; (7) inform the patient of precautions; and (8) end the medical visit. The system then summarizes the user’s knowledge of the disease and evaluates their performance in real time.

### Protocol of the Course

#### Participants

The participants were recruited offline from 112 TCM (5+3 integration) sophomores enrolled in the CDUTCM in 2019. Based on previous studies, a minimum sample size of 78 was required [25,26].

#### Inclusion and Exclusion Criteria

TCM (5+3 integration) students in their second year at the CDUTCM were included. The exclusion criteria were as follows: participants who (1) had received VSP or SP training; (2) had taken courses related to IMTCM, such as PTC-IMTCM and reception and clinical thinking skills; and (3) failed to adhere to the study schedule or withdrew from the study.

#### Randomization and Blinding

Using computer-generated randomization, the participants were randomly assigned to either a VSP-TCM group or a control group in a 1:1 ratio. The randomization was performed by individuals who were not in contact with the participants. The participants, staff, and investigators were blinded to the training assignments. Data analysis was deferred until all data were collected by investigators who were blinded to the outcomes.

#### Teacher Training and Eligibility

Teachers in the course group underwent standardized training 1 month before the course started. The standardized training planning was set based on the “Eight-Year Undergraduate Talent Training Guide of Traditional Chinese Medicine” issued by the CDUTCM. Subsequently, experienced clinical experts were invited to assess the teachers’ qualifications. Finally, we selected 2 teachers who were closely matched in terms of age, sex, teaching experience, and teaching style. Using a random coin toss, they were then assigned to either the VSP-TCM group or the control group.

#### Training Interventions and Data Collection

PTC-IMTCM was conducted in the second semester of the sophomore year (36 class hours, 3 class hours/week, a total of 12 weeks). Both groups had the same teaching materials, such as Microsoft PowerPoint presentations and textbooks. The VSP-TCM group received 36 class hours of VSP-TCM training. The teaching methods were as follows: (1) The teacher discussed the fundamentals of the disease, including etiology, pathogenesis, diagnosis, differential diagnosis, syndrome differentiation and treatment, and key points of history collection; (2) students logged into the VSP-TCM system, selected a disease to practice, and entered the simulated medical scene; (3) students completed the diagnosis and treatment with a VSP, following the process shown in Figure 2 within the specified time frame; (4) students repeated the exercise according to feedback from the VSP-TCM system; and (5) students voluntarily completed after-school exercises and recorded their learning time.

The control group received 36 class hours of traditional academic training, which included teacher teaching and group discussion. Specifically, the teaching methods were as follows: (1) The teacher discussed the fundamentals of the disease and syndrome, similar to the VSP-TCM group; (2) students were randomly assigned to several study groups; (3) students discussed the case provided by the teacher, made a diagnosis, and suggested treatments for the disease within the specified time frame; (4) the teacher evaluated the answers and provided feedback to the students; and (5) students voluntarily completed after-school exercises and recorded their learning time.

This study was initiated on February 26, 2020, and concluded on August 20, 2021. From the start of the study to March 2021, staff not involved in the study conducted a prequestionnaire investigation and gathered data on the participants’ performance in basic TCM and WM courses. Next, the 13-week PTC-IMTCM course was conducted from April 1 to July 10, 2021 (first 12 weeks, training; 13th week, evaluation). Finally, investigators who did not take part in the study collected and analyzed the data by August 20, 2021.

### Evaluation of Training Effectiveness

#### Formative Evaluation

The mini-clinical evaluation exercise (mini-CEX), a tool developed by the American College of Internal Medicine in 1995 based on the traditional clinical evaluation exercise, has teaching functions and was used to assess the residents’ clinical ability [27]. Mini-CEX has several advantages, including direct observation, a simple form, a key evaluation, and real-time feedback [28]. According to a 9-point evaluation system, the scale comprehensively evaluates the clinical competence of residents based on 4 aspects: history collection ability, physical examination ability, clinical judgment ability, and overall ability [27]. To better evaluate the students’ clinical ability, we slightly modified the mini-CEX according to Chen et al [29]. The modified mini-CEX can evaluate clinical competence based on 5 aspects: medical interviewing ability, physical examination ability, clinical judgment ability, disease treatment ability, and comprehensive ability. Multimedia Appendix 1 shows the modified mini-CEX and its usage for formative evaluation.

#### Summative Assessment

After the course, students were given 1 week to review for the assessment, and the summative assessment was conducted on a day in week 13. The summative assessment was divided into online and offline assessments. Students completed the systematic knowledge test of PTC-IMTCM online before entering the clinical skill center of the CDUTCM for the offline assessment. The offline assessment included the following steps: (1) collecting the medical history from OSPs, (2) medical writing, and (3) syndrome differentiation and treatment.

##### Online Systematic Knowledge Test

After the course, students completed an objective and standardized online case-based examination. They had 90 minutes to complete 6 cases. For the first 5 cases, there were 5 multiple-choice questions in each case, and students were required to select the right diagnosis, syndrome, treatment principle, treatment method, and prescription. Each multiple-choice question was worth 2 points. The sixth case was a case analysis question. Students were required to provide a TCM diagnosis (5 points), syndrome differentiation (5 points), a syndrome differentiation basis (15 points), treatment (5 points), a prescription (5 points), and prescription analysis (15 points) for the patient in the case. The examination had a total score of 100.

##### Offline Clinical Skill Test

Following the online examination, students proceeded to the clinical skill center of the CDUTCM for a 45-minute offline clinical skill test. For this test, we used previously developed offline clinical skill assessment methods [8]. Students encountered OSPs that we had previously trained during testing at the site. Students had 15 minutes to complete the medical history collection process, including a medical interview and physical examination. They were then given 30 minutes to complete medical records and treatment based on syndrome differentiation.

###### Scores for the Application of TCM Technology

In the assessment process, a TCM professional who was not part of the research team and did not participate in activities such as teaching and proposition scored the students’ clinical skills using a pre-established checklist. The checklist included the following items: introduction (4 points), chief complaint (8 points), current medical history (30 points), past medical history (12 points), personal medical history (12 points), family medical history (8 points), physical examination and 4 TCM examinations (16 points), and summary (10 points).

###### Scores for Written Medical Records

The medical writing checklist included the following items: general information (3 points), chief complaint (5 points), current medical history (30 points), past medical history (10 points), personal medical history (10 points), family medical history (6 points), physical examination (20 points), and 4 TCM examinations (16 points). Individual item scores were calculated and processed.

###### Scores for TCM Syndrome Differentiation and Therapeutic Regimen

The following were items were included in the checklist for TCM treatment based on syndrome differentiation: TCM diagnosis (6 points); basis for TCM diagnosis (6 points); WM diagnosis (6 points); basis for WM diagnosis (14 points); TCM syndrome type (10 points); analysis of TCM syndrome differentiation (24 points); TCM treatment method (8 points); formula (8 points); medication, administration method, and corresponding dosage (14 points); and medical advice (4 points). The examination was scored using predetermined standards.

###### OSP Real-Time Assessment Scores

Following each interaction, OSPs used the Arizona Clinical Interviewing Rating (ACIR) scale to assess students’ interpersonal communication and interview skills. The ACIR scale is a 20-item scale, with points ranging from 1 to 5 points, with 5 being the highest [30].

### Postcourse Feedback Questionnaire

A previous study’s [8] questionnaire was modified for a new audience. After the course, a survey was administered to both groups to assess students’ attitudes toward the course, command of knowledge, and proficiency in clinical skills to help us optimize the course. In addition, we conducted a questionnaire investigation among 15 teachers responsible for PTC-IMTCM to assess the potential impact of VSP-TCM on their work.

### Statistical Analysis

A blinded research analyst conducted the test. The intraclass correlation coefficient (ICC) was calculated to determine the consistency of the Mini-CEX scores. Statistical analysis was conducted using SPSS 25.0 (IBM). Continuous variables were expressed as the mean (SD) and categorical variables as a frequency or percentage. The Kolmogorov-Smirnov test was used to determine the normality of all the data. When data had a normal distribution, the independent-samples *t* test was used, otherwise the Mann-Whitney U test was used. The chi-squared (χ^2^) test was used to compare proportions. *P*≤0.05 indicated significant differences.

## Results

### Prequestionnaire Investigation

A total of 1628 questionnaires were administered to students majoring in TCM at the CDUTCM who had completed the PTC-IMTCM course; however, only 1294 (79.4%) valid questionnaires were included in the study. Despite having systematically learned PTC-IMTCM, students remained concerned regarding their clinical skills. Of 1294 students, 1096 (84.7%) believed it was necessary to improve their clinical ability. In addition, 951 (73.5%) students were willing to try VSP-TCM (Table 1).

**Table 1:** Results of the prequestionnaire investigation (N=1294).

Questions		Options, n (%)				
		Strongly agree	Agree	Neutral	Disagree	Strongly disagree
**Basic information**						
	You already have good TCM^a^ thinking.	65 (5.0)	326 (25.2)	241 (18.6)	635 (49.1)	27 (2.1)
	You are confident to handle clinical diagnosis and treatment.	122 (9.4)	351 (27.1)	218 (16.9)	507 (39.2)	96 (7.4)
	It is still necessary to significantly upgrade your clinical capacity.	558 (43.1)	538 (41.6)	147 (11.4)	43 (3.3)	8 (0.6)
**Learning methods**						
	You prefer to learn clinical skills through situation simulation rather than the teacher teaching.	275 (21.3)	699 (54.0)	158 (12.2)	91 (7.0)	71 (5.5)
	You hope to have more ways to improve your clinical skills after class.	427 (33.0)	790 (61.1)	43 (3.3)	25 (1.9)	9 (0.7)
**Learning habits**						
	You will preview clinical skills independently before the course.	278 (21.5)	507 (39.2)	138 (10.7)	277 (21.4)	94 (7.3)
	You will review clinical skills independently after the course.	329 (25.4)	512 (39.6)	147 (11.4)	224 (17.3)	82 (6.3)
**Learning interests**						
	Do you think the current clinical competence enhancement course is interesting enough that you can devote yourself to classroom learning?	91 (7.0)	102 (7.9)	342 (26.4)	575 (44.4)	184 (14.2)
**Acceptance and demand for VSP-TCM^b^**						
	You have a good understanding of virtual simulation technology.	14 (1.1)	96 (7.4)	230 (17.8)	787 (60.8)	167 (12.9)
	You are willing to try VSP-TCM teaching if there is a chance.	442 (34.2)	509 (39.3)	164 (12.7)	111 (8.6)	68 (5.3)

^a^TCM: traditional Chinese medicine.

^b^VSP-TCM: virtual standardized patient of traditional Chinese medicine.

### Student Data

A total of 84/112 (75%) students participated in this study and were randomly assigned to the VSP-TCM group (n=42, 50%) and the control group (n=42, 50%). There was no significant difference in age (*P*=.11) and sex (*P*=.64) between the 2 groups. At baseline, there was no significant difference between the 2 groups in TCM basic courses (*P*=.74), WM basic courses (*P*=.31), or the grade point average (*P*=.33); see Table 2.

**Table 2:** Baseline characteristics (N=84).

Characteristics			Training condition		
			VSP-TCM^a^ group (n=42)		Control group (n=42)
Age (years), mean (SD); *P*=.11			20.69 (0.67)		20.85 (0.60)
**Sex, n (%)**					
	Female	28 (67)		30 (71)	
	Male	14 (33)		12 (29)	
Basic courses of TCM^b^ (points), mean (SD); *P*=.74			82.85 (6.53)		83.63 (6.37)
Basic courses of WM^c^ (points), mean (SD); *P*=.31			82.57 (3.87)		82.59 (4.56)
Grade point average, mean (SD); *P*=.33			3.27 (0.33)		3.24 (0.37)

^a^VSP-TCM: virtual standardized patient of traditional Chinese medicine.

^b^TCM: traditional Chinese medicine.

^c^WM: Western medicine.

### Evaluation of Training Effectiveness

#### Formative Evaluation

**Figure 3:**
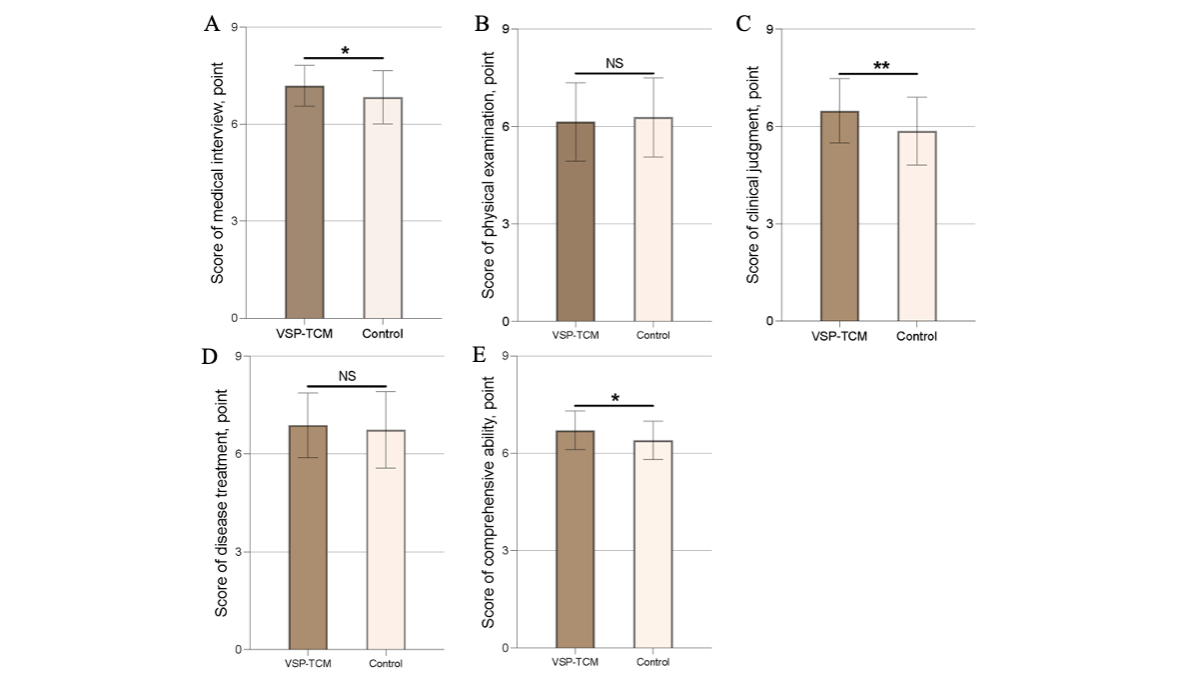
Results of formative evaluation. NS: not significant; VSP-TCM: virtual standardized patient of traditional Chinese medicine. *P<.05, **P<.01. A: Score of medical interview; B: Score of physical examination; C: Score of clinical judgment; D: Score of disease treatment; E: Score of comprehensive ability.

Figure 3 shows the mini-CEX results for both groups. The consistency among physicians responsible for evaluating students’ performance was high (ICC=0.82). After 6 weeks of training, the VSP-TCM group gained higher scores in medical interviewing ability (mean 7.19, SD 0.63, vs mean 6.83, SD 0.81; U=656.5, *P=*.04), clinical judgment (mean 6.48, SD 0.98, vs mean 5.86, SD 1.04; U=590, *P*=.006), and comprehensive ability (mean 6.71, SD 0.59, vs mean 6.40, SD 0.58; U=634.5, *P*=.02) than the control group. However, the VSP-TCM group did not show the expected advantages in physical examination (mean 6.14, SD 1.19, vs mean 6.29, SD 1.20; U=830, *P*=.64) and disease treatment (mean 6.88, SD 0.98, vs mean 6.74, SD 1.16; U=827, *P*=.62), and the score was slightly lower than that of the control group.

#### Summative Assessment

##### Online Systematic Knowledge Test

**Figure 4:**
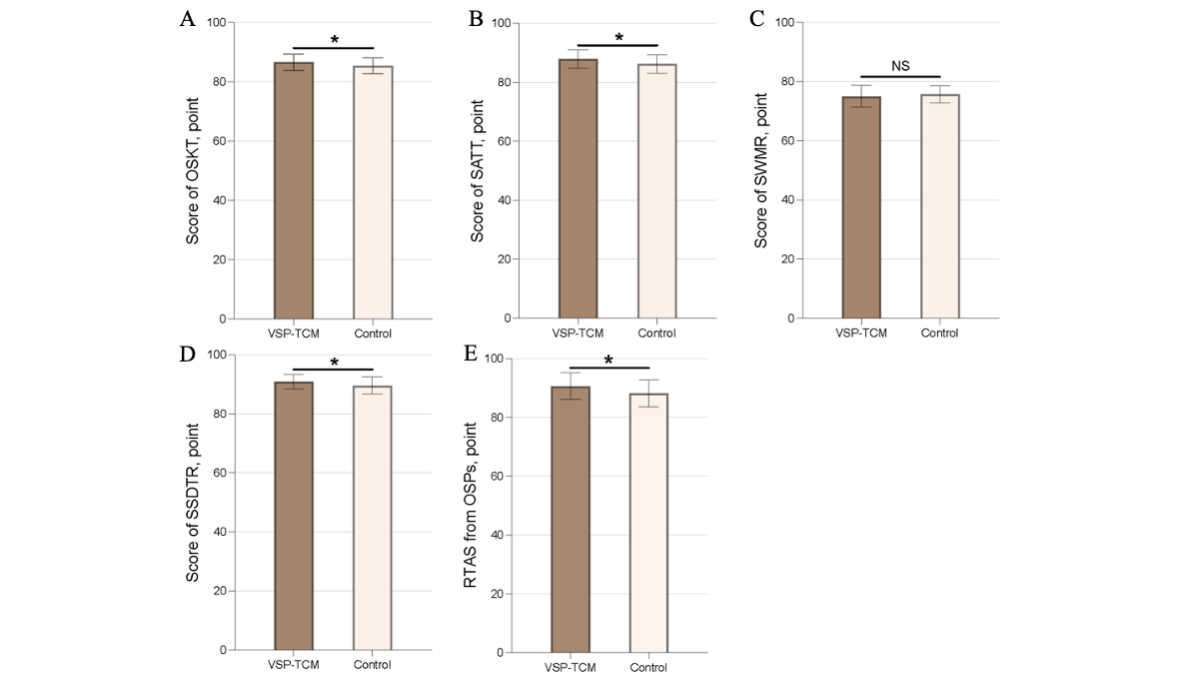
Scores of summative assessment (*P<.05). OSKT: online systematic knowledge test (A); SATT: scores for the application of traditional Chinese medicine technology (B); SWMR: scores for written medical records (C); SSDTR: scores for TCM syndrome differentiation and therapeutic regimen (D); RTAS: Real-Time Assessment Scores (E); OSP: occupational standardized patient (E); VSP-TCM: virtual standardized patient of traditional Chinese medicine.

After 12 weeks of the course, the VSP-TCM group mastered the systematic knowledge of the course better than the control group (Figure 4A). In the OSKT, the VSP-TCM group scored higher than the control group (mean 86.62, SD 2.71, vs mean 85.38, SD 2.62; U=660.5, *P*=.046).

##### Offline Clinical Skill Test

###### Scores for the Application of TCM Technology

The VSP-TCM group outperformed the control group in receiving VSP-TCM. The VSP-TCM group performed better in the application of TCM skills (mean 87.86, SD 3.04, vs mean 86.19, SD 3.08; t_82_=2.464, *P*=.02, d=82); see Figure 4B.

###### Scores for Written Medical Records

VSP-TCM did not provide the expected benefits in improving students’ ability to write medical records. The VSP-TCM group scored lower than the control group (mean 75.07, SD 3.61, vs mean 75.71, SD 2.86; t_82_=0.8945, *P*=.37, d=82); see Figure 4C.

###### Scores for TCM Syndrome Differentiation and Therapeutic Regimen

VSP-TCM effectively improved core TCM skills, including syndrome differentiation and treatment. The VSP-TCM group had higher scores for TCM syndrome differentiation and treatment than the control group (mean 90.93, SD 2.42, vs mean 89.60, SD 2.86; U=636, *P*=.03); see Figure 4D.

###### Real-Time Assessment Scores From OSPs

VSP-TCM had a satisfactory effect on improving students’ interpersonal communication and interview skills. The real-time evaluation score from OSPs for the VSP-TCM group was significantly higher than the control group (mean 90.67, SD 4.52, vs mean 88.24, SD 4.56; U=618.5, *P*=.02); see Figure 4E.

### Postcourse Feedback Questionnaire

Table 3 displays the results of the postcourse feedback questionnaire administered to students. Of the 11 items in the questionnaire, 6 (55%) showed significant differences between both groups. Of 42 students in the VSP-TCM group, 39 (93%) believed that the course improved their TCM thinking ability as opposed to 37 (88%) students in the control group (*P*=.002). Regarding medical history collection, 38 (91%) students in the VSP-TCM group found the course beneficial as opposed to 30 (71%) students in the control group (*P*=.001). In syndrome differentiation and treatment, and critical thinking ability, 38 (91%) students in the VSP-TCM group found VSP-TCM helpful, while 37 (88%) students in the control group found traditional academic training helpful (*P*=.046). In addition, 40 (95%) students in the VSP-TCM group gained better comprehensive clinical application ability as opposed to 36 (86%) students in the control group (*P*=.009). Furthermore, 36 (86%) students trained with VSP-TCM grasped better interpersonal communication skills, whereas only 28 (67%) students in the control group grasped the same skills (*P*=.01). Interestingly, 37 (88%) students who received VSP-TCM teaching significantly improved their autonomous learning ability; however, only 28 (67%) students in the control group endorsed this viewpoint (*P*=.01). Overall, students in both groups were satisfied with the course (*P*=.23); however, VSP-TCM did not improve their medical writing ability compared to traditional academic training (*P*=.13).

Table 4 shows the results of the feedback questionnaire administered to teachers. Overall, we found that teachers held a positive perspective on VSP-TCM. No less than 12 (80%) of 15 teachers expressed their belief that VSP-TCM has the potential to become a prominent trend in TCM education and expressed their willingness to integrate it into their teaching practices. Simultaneously, they recognized the benefits of using VSP-TCM to enhance students’ motivation for learning (n=12, 80%), regarding it as a valuable adjunct to bedside instruction (n=15, 100%). Furthermore, it is worth mentioning that teachers preferred to develop VSPs based on syndromes (n=14, 93%) rather than diseases (n=11, 73%), which highlighted the characteristics of “treatment based on syndrome differentiation.” Finally, all teachers unanimously agreed that VSP-TCM has the potential to enhance teaching efficiency, while effectively minimizing teaching expenses, thus establishing it as a cost-effective instructional resource.

**Table 3:** Results of postcourse feedback questionnaire administration to students (N=84).

Questions	VSP-TCM^a^ group (n=42), n (%)						Control group (n=42), n (%)						*P* value
	Strongly agree	Agree	Neutral	Disagree	Strongly disagree	Strongly agree		Agree	Neutral	Disagree	Strongly disagree		
You are satisfied with the course.	23 (55)	17 (40)	1 (2)	1 (2)	0	15 (36)		20 (48)	5 (12)	1 (2)	1 (2)	.23	
The course improved your interest in learning TCM^b^.	17 (40)	21 (50)	3 (7)	1 (2)	0	12 (29)		20 (48)	7 (17)	2 (5)	1 (2)	.43	
The course enhanced your confidence in dealing with clinical work.	11 (26)	19 (45)	8 (19)	3 (7)	1 (2)	6 (14)		21 (50)	12 (29)	2 (5)	1 (2)	.63	
The course enhanced your TCM thinking ability.	32 (76)	7 (17)	2 (5)	1 (2)	0	15 (36)		22 (5)	4 (10)	1 (2)	0	.002	
The course consolidated the basic theoretical knowledge you have learned before.	31 (74)	7 (17)	4 (10)	0	0	27 (64)		13 (31)	2 (5)	0	0	.25	
The course enhanced your ability to collect medical history.	28 (67)	10 (24)	4 (10)	0	0	10 (24)		20 (48)	8 (19)	4 (10)	0	.001	
The course enhanced your ability to write medical records.	12 (29)	12 (29)	13 (31)	4 (10)	1 (2)	10 (24)		23 (55)	7 (17)	2 (5)	0	.13	
The course enhanced your syndrome differentiation and treatment ability, and critical thinking.	25 (60)	13 (31)	1 (2)	2 (5)	1 (2)	12 (29)		25 (60)	3 (7)	1 (2)	1 (2)	.046	
The course enhanced your ability to construct harmonious doctor-patient relationships and develop interpersonal communication skills.	20 (48)	16 (38)	4 (10)	2 (5)	0	8 (19)		20 (48)	13 (31)	1 (2)	0	.01	
The course enhanced your comprehensive clinical application ability.	12 (29)	28 (67)	1 (2)	1 (2)	0	6 (14)		25 (60)	11 (26)	0	0	.009	
The course enhanced your ability to self-study.	25 (60)	12 (29)	3 (7)	1 (2)	1 (2)	10 (24)		18 (43)	11 (26)	2 (5)	1 (2)	.01	

^a^VSP-TCM: virtual standardized patients of traditional Chinese medicine.

^b^TCM: traditional Chinese medicine.

**Table 4:** Results of feedback questionnaire administration to teachers.

Questions	Options, n (%)				
	Strongly agree	Agree	Neutral	Disagree	Strongly disagree
Do you agree that VSP-TCM^a^ can become a trend in TCM^b^ education?	0	12 (80)	3 (20)	0	0
Are you willing to use VSP-TCM in the PTC-IMTCM^c^ course?	6 (40)	7 (47)	2 (13)	0	0
Do you agree that VSP-TCM can enhance teaching efficiency?	1 (7)	14 (93)	0	0	0
Do you agree that VSP-TCM can reduce teaching costs?	8 (53)	7 (47)	0	0	0
Would you consider using VSP-TCM as a supplement to bedside teaching?	9 (60)	6 (40)	0	0	0
Do you agree that VSP-TCM can reduce students’ reliance on real patients when training in clinical skills?	0	10 (67)	5 (33)	0	0
Do you agree that VSP-TCM can improve students’ interest in learning TCM?	0	12 (80)	3 (20)	0	0
Do you agree that VSP-TCM should be constructed based on different diseases?	4 (27)	7 (47)	4 (27)	0	0
Do you agree that VSP-TCM should be constructed based on different syndromes?	3 (20)	11 (73)	1 (7)	0	0
Do you agree that VSP-TCM can enhance students’ ability to conduct medical interviews?	3 (20)	10 (67)	2 (13)	0	0
Do you agree that VSP-TCM can enhance students’ TCM clinical skills, such as tongue and pulse diagnosis?	2 (13)	9 (60)	3 (20)	1 (7)	0
Do you agree that VSP-TCM can enhance students’ medical writing ability?	1 (7)	6 (40)	6 (40)	2 (13)	0
Do you agree that VSP-TCM can enhance students’ syndrome differentiation and treatment ability?	1 (7)	7 (47)	5 (33)	2 (13)	0
Do you agree that VSP-TCM can enhance students’ interpersonal communication skills?	1 (7)	8 (53)	6 (40)	0	0

^a^VSP-TCM: virtual standardized patient of traditional Chinese medicine.

^b^TCM: traditional Chinese medicine.

^c^PTC-IMTCM: practical training course of internal medicine of traditional Chinese medicine.

### Discussion

#### Principal Findings

This study demonstrated that compared with traditional academic training, VSP-TCM significantly improves abilities in medical interviewing, clinical judgment, TCM technology application, and systematic knowledge among TCM students. The VSP-TCM system enabled TCM education to be more efficient and less stressful for teachers. It was also helpful for reforming the course and achieving the goal of training applied talents.

IMTCM is a core course that helps students learn how to diagnose and treat patients using TCM technology in the real world [31]. PTC-IMTCM is a supplemental course of IMTCM that aims to enhance students’ sense of innovation [32]. However, strict rules on medical ethics, abstract teaching content, and high costs make it challenging to improve teaching efficacy [33,34]. Therefore, there is an urgent need for a solution that can overcome the current dilemma. Virtual simulation and SPs are mature technologies that have been widely used in medical education [35,36]. VSPs are low-cost, risk-free alternatives [37]. We conducted a questionnaire survey before starting this study, and the findings revealed that medical students hope that the current methods of the education curriculum are innovatively reformed. The CDUTCM launched the VSP-TCM project in the autumn semester of 2019. In the spring semester of 2020, we cooperated with Shanghai Dream Road Digital Technology Co., Ltd. to design the system. The project was built and implemented in the autumn of 2020.

This randomized controlled trial compared the teaching efficacy of VSP-TCM in the PTC-IMTCM course and traditional academic training for 3 months. The findings supported the merits of VSP-TCM; students faced less stress in the simulative clinic, allowing them to devote more time to learning. Through repeated practice within a safe environment, they gradually became familiar with the diagnosis and treatment process, in line with the findings of a previous study [38]. The results of the mini-CEX demonstrated that in the middle of the semester, students’ comprehensive ability, including medical interviewing and clinical judgment, significantly improved compared to the control group. Furthermore, results of the summative assessment indicated that VSP-TCM improves students’ theoretical knowledge, medical history collection skills, and syndrome differentiation and treatment ability. Compared to traditional academic training, students’ interpersonal communication skills significantly improved using VSP-TCM, thus enabling them to build a more harmonious doctor-patient relationship in their future work and help improve patient prognosis [39]. In addition to their clinical and comprehensive abilities, the results of the postcourse questionnaire administration revealed that VSP-TCM effectively improves students’ autonomous learning ability. This may be because VSP-TCM is more repeatable than traditional academic training, providing students with more resources and opportunities to conduct medical interviews.

VSP-TCM significantly improved 2 core TCM skills, the ability to collect medical history and the syndrome differentiation and treatment ability. Students’ mastery of the content, logic, and communication skills of inquiry reflected the improvement in their ability to collect medical history. More than 85% of students in the experimental group agreed that VSP-TCM effectively helped them improve their interpersonal communication skills, as opposed to less than 67% in the control group. The real-time evaluation of offline clinical skills from OSPs also indicated that VSP-TCM can significantly improve students’ competence. Given that a solid command of theoretical knowledge guarantees clinical ability, students in the experimental group performed better on the OSKT than the control group, indicating improvement in their theoretical knowledge. This may be attributed to 2 unique advantages of VSP-TCM: repeatability and real-time feedback. Students could better remember the content and logic of medical history collection due to repeatability. In addition, real-time feedback offered students valuable insights into the overall quality of their performance and identified areas that needed further improvement [40]. This allowed students to make appropriate adjustments in the next training to consolidate and improve their skills according to the feedback [3].

Overall, the postcourse feedback questionnaire showed that both groups were satisfied with the course. The course had positive teaching efficacy and achieved the teaching objectives. VSP-TCM promoted the development of TCM thinking and critical thinking. It also strengthened students’ clinical skills, self-study ability, and comprehensive ability to help them become qualified future doctors. Interestingly, although VSP-TCM improved students’ clinical skills, they did not have a high confidence level in solving clinical problems. This may be because the students had not yet officially begun their clinical work, which was unfamiliar to them. Furthermore, students believed that VSP-TCM did not significantly improve their medical writing ability, which contradicted our assumption. This may be because VSP-TCM automatically records and displays all operations of students, with easy access to clinical data. Consequently, the students did not develop the awareness and habit of taking notes during medical interviews. This gave us a preliminary idea for improving VSP-TCM. In addition, teachers’ perspectives on the VSP-TCM system indicated that it could be a good teaching tool, which could improve teaching efficacy and partly reduce the teaching pressure on teachers. However, the system should be more consistent with the characteristics of TCM, such as simulating patients with different syndromes instead of diseases.

VSP-TCM is cost-effective, which is an outstanding advantage. According to our previous experience, OSP and SSP systems incur substantial costs in terms of human resources and materials [8,9]. VSP-TCM is a web-based system that only requires one-time development. Students can access and practice online through the internet, while the course group only needs to allocate a small amount of funds for system updates, upgrades, and maintenance. Thus, VSP-TCM is a feasible, practical, and cost-effective alternative, and it is worthy of promotion.

#### Limitations

Although the findings of this study are positive, it has some limitations that should be acknowledged. First, in this prospective study, we only evaluated the clinical skills of students during and after the course, with no long-term follow-up. We were unaware of how VSP-TCM maintained the trainees’ competence. Second, VSP-TCM cannot convey some physical signs of real patients. For example, TCM-specific pulse conditions can only be displayed through images, preventing trainees from gaining real clinical experience in pulse diagnosis [8]. In addition, this was a single-center study, which affected the universality of the findings. Therefore, future large-scale, multicenter studies are warranted.

#### Conclusion

This randomized controlled trial demonstrated the feasibility of VSP-TCM in improving the clinical competence of medical students. VSP-TCM may be a replacement for OSPs and SSPs in universities with limited expenditure. VSP-TCM deserves more attention and promotion. Therefore, future studies aiming to improve VSP-TCM systems that can effectively enhance the clinical abilities of medical students are warranted.

## Appendix

Multimedia Appendix 1 Modified mini-clinical evaluation exercise (mini-CEX) and the flow of formative evaluation.

Multimedia Appendix 2 Consolidated Standards of Reporting Trials of Electronic and Mobile Health Applications and Online Telehealth (CONSORT-eHEALTH) checklist (V 1.6.1).

## Abbreviations

ACIR: Arizona Clinical Interviewing Rating
CDUTCM: Chengdu University of Traditional Chinese Medicine
ICC: intraclass correlation coefficient
IMTCM: internal medicine of traditional Chinese medicine
mini-CEX: mini-clinical evaluation exercise
OSKT: online systematic knowledge test
OSP: occupational standardized patient
PTC-IMTCM: practical training course of internal medicine of traditional Chinese medicine
SP: standardized patient
SSP: student standardized patient
TCM: traditional Chinese medicine
VSP: virtual standardized patient
WM: Western medicine

## Data Availability

The “Eight-Year Undergraduate Talent Training Guide of Traditional Chinese Medicine” issued by the Chengdu University of Traditional Chinese Medicine is available upon reasonable request.
