# Supplementary material for: Virtual Standardized Patients Versus Traditional Academic Training for Improving Clinical Competence Among Traditional Chinese Medicine Students: Prospective Randomized Controlled Trial
Source: J Med Internet Res. 2023 Sep 20;25:e43763. doi: 10.2196/43763 (PMC10551797; doi:10.2196/43763)
Supplement: Multimedia Appendix 1 [file jmir_v25i1e43763_app1.docx]

**MULTIMEDIA APPENDIX 1**

**Supplementary Table S1** Modified Mini-CEX

| **Domain** | **Content** | **Score** |
| --- | --- | --- |
| Medical interview ability | Proper addressing of patients and comfortable self introduction | 1 |
|  | The priority of the inquiry is clear, and the patient's chief complaint can be accurately summarized | 2 |
|  | The characteristics of the main clinical symptoms were inquired in detail (for example, the patients with pain as the main complaint should be inquired about their pain location, nature, duration, attack frequency, aggravating and relieving factors, etc.) | 2 |
|  | The general conditions (including the patient's diet, stool, sleep, spirit, weight, etc.) were inquired in detail | 1 |
|  | Asked about the past history in detail | 1 |
|  | Asked about personal history in detail | 1 |
|  | Asked about the family history in detail | 1 |
| physical examination ability | Protect patient privacy | 1 |
|  | Inform the patient of the purpose of the physical examination | 1 |
|  | The articles required for physical examination are complete | 1 |
|  | Wash hands regularly before and after contacting patients | 1 |
|  | Assist the patient in taking the correct position | 1 |
|  | The physical examination should be standardized, with moderate strength, and pay attention to humanistic care (such as covering the stethoscope with both hands) | 2 |
|  | The physical examination sequence is reasonable, and the patient's position is not changed repeatedly | 1 |
|  | The physical examination system is comprehensive, and no inspection items are omitted (such as tongue examination and pulse examination in TCM) | 1 |
| clinical judgment ability | Correct diagnosis by western medicine | 1 |
|  | Correct differential diagnosis by western medicine | 2 |
|  | Correct differentiation of diseases in TCM | 1 |
|  | Correct syndrome differentiation of TCM | 1 |
|  | Correct differential diagnosis of TCM | 2 |
|  | Correct analysis of TCM syndrome differentiation | 2 |
| disease treatment ability | The principle of western medicine treatment is accurate | 1 |
|  | Accurately arrange further auxiliary inspections | 1 |
|  | Correct treatment of TCM | 2 |
|  | Correct TCM Prescription | 1 |
|  | Explain the decoction and administration of TCM, precautions and possible body reactions (including efficacy and side effects) after taking the medicine to patients | 2 |
|  | Correctly analyze the TCM prescriptions | 2 |
| comprehensive ability | Ability to accurately judge the severity of illness | 3 |
|  | Ability to deal with emergencies | 3 |
|  | Complete the assessment within the specified time | 2 |
|  | Overall efficiency | 1 |

**Supplementary file S1** Formative evaluation flow

1. After the sixth course teaching, all the students were assigned to each clinical department of the Hospital of Chengdu University of Traditional Chinese Medicine for practice by using the random number method. The selected clinical departments include respiratory department (disease: cough), cardiology department (disease: palpitation), neurology department (disease: headache), digestive department (disease: stomach pain), nephrology department (disease: edema), and endocrinology department (disease: thirst).
2. Considering that the evaluation results of Mini CEX scale are easily affected by the subjective factors of different evaluators, we conducted systematic training for residents in the above departments in advance to minimize the subjective bias caused by the evaluation of different doctors. After the training, a resident who did not participate in the evaluation would receive a standardized patient, and the trained resident will score the diagnosis and treatment according to the modified Mini-CEX. We calculated intragroup correlation coefficients (ICC) to evaluate the consistency among residents. When ICC>0.75, we thought the reliability among residents was high, and agreed they to participate in the following evaluation.
3. Each student carried out systematic diagnosis and treatment activities for patients, including medical history collection, physical examination, clinical diagnosis and development of diagnosis and treatment plan (including auxiliary examination, western medicine treatment, and syndrome differentiation and treatment). A systematically trained resident physician was present to evaluate the clinical ability of the interns according to the modified Mini-CEX scale and gave the student real-time feedback.
4. Calculate scores of each fields of Mini-CEX of the two groups.
