# Supplementary material for: Virtual Standardized Patients Versus Traditional Academic Training for Improving Clinical Competence Among Traditional Chinese Medicine Students: Prospective Randomized Controlled Trial
Source: J Med Internet Res. 2023 Sep 20;25:e43763. doi: 10.2196/43763 (PMC10551797; doi:10.2196/43763)
Supplement: Multimedia Appendix 2 [file jmir_v25i1e43763_app2.pdf]

CONSORT-EHEALTH (V 1.6.1) - Submission/Publication Form

The CONSORT-EHEALTH checklist is intended for authors of randomized trials evaluating web-based and Internet-based applications/interventions, including mobile interventions, electronic games (incl multiplayer games), social media, certain telehealth applications, and other interactive and/or networked electronic applications. Some of the items (e.g. all subitems under item 5 - description of the intervention) may also be applicable for other study designs.

The goal of the CONSORT EHEALTH checklist and guideline is to be
a) a guide for reporting for authors of RCTs,
b) to form a basis for appraisal of an ehealth trial (in terms of validity)

CONSORT-EHEALTH items/subitems are MANDATORY reporting items for studies published in the Journal of Medical Internet Research and other journals / scientific societies endorsing the checklist.

Items numbered 1., 2., 3., 4a., 4b etc are original CONSORT or CONSORT-NPT (non-pharmacologic treatment) items.
Items with Roman numerals (i., ii, iii, iv etc.) are CONSORT-EHEALTH extensions/clarifications.

As the CONSORT-EHEALTH checklist is still considered in a formative stage, we would ask that you also RATE ON A SCALE OF 1-5 how important/useful you feel each item is FOR THE PURPOSE OF THE CHECKLIST and reporting guideline (optional).

Mandatory reporting items are marked with a red *.
In the textboxes, either copy & paste the relevant sections from your manuscript into this form - please include any quotes from your manuscript in QUOTATION MARKS,
or answer directly by providing additional information not in the manuscript, or elaborating on why the item was not relevant for this study.

YOUR ANSWERS WILL BE PUBLISHED AS A SUPPLEMENTARY FILE TO YOUR PUBLICATION IN JMIR AND ARE CONSIDERED PART OF YOUR PUBLICATION (IF ACCEPTED).
Please fill in these questions diligently. Information will not be copyedited, so please use proper spelling and grammar, use correct capitalization, and avoid abbreviations.

DO NOT FORGET TO SAVE AS PDF _AND_ CLICK THE SUBMIT BUTTON SO YOUR ANSWERS ARE IN OUR DATABASE !!!

Citation Suggestion (if you append the pdf as Appendix we suggest to cite this paper in the caption):
Eysenbach G, CONSORT-EHEALTH Group
CONSORT-EHEALTH: Improving and Standardizing Evaluation Reports of Web-based and Mobile Health Interventions
J Med Internet Res 2011;13(4):e126

URL: http://www.jmir.org/2011/4/e126/
doi: 10.2196/jmir.1923
PMID: 22209829

登录 Google 即可保存进度。了解详情

* 表示必填

Your name *
First Last

Jinhao

Primary Affiliation (short), City, Country *
University of Toronto, Toronto, Canada

Hospital of Chengdu University of Traditional C

Your e-mail address *
abc@gmail.com

zengjinhao@cdutcm.edu.cn

Title of your manuscript *
Provide the (draft) title of your manuscript.

Virtual Standardized Patients versus Academic Training for Improving Clinical Competence among Traditional Chinese Medicine Students: A Prospective Randomized Trial

Name of your App/Software/Intervention *

If there is a short and a long/alternate name, write the short name first and add the long name in brackets.

VSP-TCM

Evaluated Version (if any)

e.g. "V1", "Release 2017-03-01", "Version 2.0.27913"

您的回答

Language(s) *

What language is the intervention/app in? If multiple languages are available, separate by comma (e.g. "English, French")

Chinese

URL of your Intervention Website or App

e.g. a direct link to the mobile app on app in appstore (itunes, Google Play), or URL of the website. If the intervention is a DVD or hardware, you can also link to an Amazon page.

您的回答

URL of an image/screenshot (optional)

您的回答

Accessibility *

Can an enduser access the intervention presently?

- ○ access is free and open
- ◉ access only for special usergroups, not open
- ○ access is open to everyone, but requires payment/subscription/in-app purchases
- ○ app/intervention no longer accessible
- ○ 其他:

Primary Medical Indication/Disease/Condition *

e.g. "Stress", "Diabetes", or define the target group in brackets after the condition, e.g. "Autism (Parents of children with)", "Alzheimers (Informal Caregivers of)"

Medical education

Primary Outcomes measured in trial *

comma-separated list of primary outcomes reported in the trial

Summative Assessment

Secondary/other outcomes

Are there any other outcomes the intervention is expected to affect?

您的回答

Recommended "Dose" *

What do the instructions for users say on how often the app should be used?

- ○ Approximately Daily
- ◉ Approximately Weekly
- ○ Approximately Monthly
- ○ Approximately Yearly
- ○ "as needed"
- ○ 其他:

Approx. Percentage of Users (starters) still using the app as recommended after 3 months *

- ◉ unknown / not evaluated
- ○ 0-10%
- ○ 11-20%
- ○ 21-30%
- ○ 31-40%
- ○ 41-50%
- ○ 51-60%
- ○ 61-70%
- ○ 71%-80%
- ○ 81-90%
- ○ 91-100%
- ○ 其他:

Overall, was the app/intervention effective? *

- ( ) yes: all primary outcomes were significantly better in intervention group vs control
- (•) partly: SOME primary outcomes were significantly better in intervention group vs control
- ( ) no statistically significant difference between control and intervention
- ( ) potentially harmful: control was significantly better than intervention in one or more outcomes
- ( ) inconclusive: more research is needed
- ( ) 其他:

Article Preparation Status/Stage *

At which stage in your article preparation are you currently (at the time you fill in this form)

- ( ) not submitted yet - in early draft status
- ( ) not submitted yet - in late draft status, just before submission
- ( ) submitted to a journal but not reviewed yet
- (•) submitted to a journal and after receiving initial reviewer comments
- ( ) submitted to a journal and accepted, but not published yet
- ( ) published
- ( ) 其他:

Journal *

If you already know where you will submit this paper (or if it is already submitted), please provide the journal name (if it is not JMIR, provide the journal name under "other")

- ○ not submitted yet / unclear where I will submit this
- ◉ Journal of Medical Internet Research (JMIR)
- ○ JMIR mHealth and UHealth
- ○ JMIR Serious Games
- ○ JMIR Mental Health
- ○ JMIR Public Health
- ○ JMIR Formative Research
- ○ Other JMIR sister journal
- ○ 其他:

Is this a full powered effectiveness trial or a pilot/feasibility trial? *

- ◉ Pilot/feasibility
- ○ Fully powered

Manuscript tracking number *

If this is a JMIR submission, please provide the manuscript tracking number under "other" (The ms tracking number can be found in the submission acknowledgement email, or when you login as author in JMIR. If the paper is already published in JMIR, then the ms tracking number is the four-digit number at the end of the DOI, to be found at the bottom of each published article in JMIR)

- ○ no ms number (yet) / not (yet) submitted to / published in JMIR
- ◉ 其他: #43763

TITLE AND ABSTRACT

1a) TITLE: Identification as a randomized trial in the title

1a) Does your paper address CONSORT item 1a? *

I.e does the title contain the phrase "Randomized Controlled Trial"? (if not, explain the reason under "other")

- (selected) yes
- 其他:

1a-i) Identify the mode of delivery in the title

Identify the mode of delivery. Preferably use “web-based” and/or “mobile” and/or “electronic game” in the title. Avoid ambiguous terms like “online”, “virtual”, “interactive”. Use “Internet-based” only if Intervention includes non-web-based Internet components (e.g. email), use “computer-based” or “electronic” only if offline products are used. Use “virtual” only in the context of “virtual reality” (3-D worlds). Use “online” only in the context of “online support groups”. Complement or substitute product names with broader terms for the class of products (such as “mobile” or “smart phone” instead of “iphone”), especially if the application runs on different platforms.

subitem not at all important

- 1
- 2
- 3
- 4 (selected)
- 5

essential

清除所选内容

Does your paper address subitem 1a-i? *

Copy and paste relevant sections from manuscript title (include quotes in quotation marks "like this" to indicate direct quotes from your manuscript), or elaborate on this item by providing additional information not in the ms, or briefly explain why the item is not applicable/relevant for your study

Yes. "A Prospective Randomized Trial."

1a-ii) Non-web-based components or important co-interventions in title

Mention non-web-based components or important co-interventions in title, if any (e.g., “with telephone support”).

subitem not at all important

1 ○

2 ○

3 ◉

4 ○

5 ○

essential

清除所选内容

Does your paper address subitem 1a-ii?

Copy and paste relevant sections from manuscript title (include quotes in quotation marks "like this" to indicate direct quotes from your manuscript), or elaborate on this item by providing additional information not in the ms, or briefly explain why the item is not applicable/relevant for your study

您的回答

1a-iii) Primary condition or target group in the title

Mention primary condition or target group in the title, if any (e.g., “for children with Type I Diabetes”) Example: A Web-based and Mobile Intervention with Telephone Support for Children with Type I Diabetes: Randomized Controlled Trial

subitem not at all important

1 ○

2 ○

3 ○

4 ○

5 ◉

essential

清除所选内容

Does your paper address subitem 1a-iii? *

Copy and paste relevant sections from manuscript title (include quotes in quotation marks "like this" to indicate direct quotes from your manuscript), or elaborate on this item by providing additional information not in the ms, or briefly explain why the item is not applicable/relevant for your study

Yes. "Clinical Competence among Traditional Chinese Medicine Students."

1b) ABSTRACT: Structured summary of trial design, methods, results, and conclusions

NPT extension: Description of experimental treatment, comparator, care providers, centers, and blinding status.

1b-i) Key features/functionalities/components of the intervention and comparator in the METHODS section of the ABSTRACT

Mention key features/functionalities/components of the intervention and comparator in the abstract. If possible, also mention theories and principles used for designing the site. Keep in mind the needs of systematic reviewers and indexers by including important synonyms. (Note: Only report in the abstract what the main paper is reporting. If this information is missing from the main body of text, consider adding it)

subitem not at all important

1 ○

2 ◉

3 ○

4 ○

5 ○

essential

清除所选内容

Does your paper address subitem 1b-i? *

Copy and paste relevant sections from the manuscript abstract (include quotes in quotation marks "like this" to indicate direct quotes from your manuscript), or elaborate on this item by providing additional information not in the ms, or briefly explain why the item is not applicable/relevant for your study

Yes. "To build a VSP-TCM application according to the characteristics of PTC-IMTCM and the needs of students and to compare its efficacy with traditional teaching in improving TCM clinical competence among students."

1b-ii) Level of human involvement in the METHODS section of the ABSTRACT

Clarify the level of human involvement in the abstract, e.g., use phrases like “fully automated” vs. “therapist/nurse/care provider/physician-assisted” (mention number and expertise of providers involved, if any). (Note: Only report in the abstract what the main paper is reporting. If this information is missing from the main body of text, consider adding it)

subitem not at all important

1 ○

2 ○

3 ○

4 ◉

5 ○

essential

清除所选内容

Does your paper address subitem 1b-ii?

Copy and paste relevant sections from the manuscript abstract (include quotes in quotation marks "like this" to indicate direct quotes from your manuscript), or elaborate on this item by providing additional information not in the ms, or briefly explain why the item is not applicable/relevant for your study

您的回答

1b-iii) Open vs. closed, web-based (self-assessment) vs. face-to-face assessments in the METHODS section of the ABSTRACT

Mention how participants were recruited (online vs. offline), e.g., from an open access website or from a clinic or a closed online user group (closed usergroup trial), and clarify if this was a purely web-based trial, or there were face-to-face components (as part of the intervention or for assessment). Clearly say if outcomes were self-assessed through questionnaires (as common in web-based trials). Note: In traditional offline trials, an open trial (open-label trial) is a type of clinical trial in which both the researchers and participants know which treatment is being administered. To avoid confusion, use "blinded" or "unblinded" to indicated the level of blinding instead of "open", as "open" in web-based trials usually refers to "open access" (i.e. participants can self-enrol). (Note: Only report in the abstract what the main paper is reporting. If this information is missing from the main body of text, consider adding it)

subitem not at all important

1 ○

2 ○

3 ◉

4 ○

5 ○

essential

清除所选内容

Does your paper address subitem 1b-iii?

Copy and paste relevant sections from the manuscript abstract (include quotes in quotation marks "like this" to indicate direct quotes from your manuscript), or elaborate on this item by providing additional information not in the ms, or briefly explain why the item is not applicable/relevant for your study

您的回答

1b-iv) RESULTS section in abstract must contain use data

Report number of participants enrolled/assessed in each group, the use/uptake of the intervention (e.g., attrition/adherence metrics, use over time, number of logins etc.), in addition to primary/secondary outcomes. (Note: Only report in the abstract what the main paper is reporting. If this information is missing from the main body of text, consider adding it)

subitem not at all important

1 ○

2 ○

3 ○

4 ◉

5 ○

essential

清除所选内容

Does your paper address subitem 1b-iv?

Copy and paste relevant sections from the manuscript abstract (include quotes in quotation marks "like this" to indicate direct quotes from your manuscript), or elaborate on this item by providing additional information not in the ms, or briefly explain why the item is not applicable/relevant for your study

您的回答

1b-v) CONCLUSIONS/DISCUSSION in abstract for negative trials

Conclusions/Discussions in abstract for negative trials: Discuss the primary outcome - if the trial is negative (primary outcome not changed), and the intervention was not used, discuss whether negative results are attributable to lack of uptake and discuss reasons. (Note: Only report in the abstract what the main paper is reporting. If this information is missing from the main body of text, consider adding it)

subitem not at all important

1 ○

2 ○

3 ◉

4 ○

5 ○

essential

清除所选内容

Does your paper address subitem 1b-v?

Copy and paste relevant sections from the manuscript abstract (include quotes in quotation marks "like this" to indicate direct quotes from your manuscript), or elaborate on this item by providing additional information not in the ms, or briefly explain why the item is not applicable/relevant for your study

您的回答

INTRODUCTION

2a) In INTRODUCTION: Scientific background and explanation of rationale

2a-i) Problem and the type of system/solution

Describe the problem and the type of system/solution that is object of the study: intended as stand-alone intervention vs. incorporated in broader health care program? Intended for a particular patient population? Goals of the intervention, e.g., being more cost-effective to other interventions, replace or complement other solutions? (Note: Details about the intervention are provided in “Methods” under 5)

subitem not at all important

1 ○

2 ○

3 ○

4 ◉

5 ○

essential

清除所选内容

Does your paper address subitem 2a-i? *

Copy and paste relevant sections from the manuscript (include quotes in quotation marks "like this" to indicate direct quotes from your manuscript), or elaborate on this item by providing additional information not in the ms, or briefly explain why the item is not applicable/relevant for your study

Yes. "Therefore, trained actors are incorporated as standardized patients (SPs) to not only train students for medical education but to also help reduce their anxiety, recreate the medical environment, and improve teaching efficiency by providing real-time feedback on students’ diagnoses and therapeutic activities"; "An alternative training approach could be the use of virtual standardized patient (VSP) programs, which employ computerized characters for SP encounters."

2a-ii) Scientific background, rationale: What is known about the (type of) system

Scientific background, rationale: What is known about the (type of) system that is the object of the study (be sure to discuss the use of similar systems for other conditions/diagnoses, if appropiate), motivation for the study, i.e. what are the reasons for and what is the context for this specific study, from which stakeholder viewpoint is the study performed, potential impact of findings [2]. Briefly justify the choice of the comparator.

subitem not at all important

1 ○

2 ○

3 ○

4 ○

5 ◉

essential

清除所选内容

Does your paper address subitem 2a-ii? *

Copy and paste relevant sections from the manuscript (include quotes in quotation marks "like this" to indicate direct quotes from your manuscript), or elaborate on this item by providing additional information not in the ms, or briefly explain why the item is not applicable/relevant for your study

Yes. "An alternative training approach could be the use of virtual standardized patient (VSP) programs, which employ computerized characters for SP encounters. Compared with SP, VSP has significant advantages as it requires fewer personnel and resources, is available at any time, and is highly customizable. In addition, it can offer highly interactive and engaging experiences to trainees. VSP is significantly useful in clinical scenarios where SPs are difficult to employ; it facilitates effective communication between doctors and patients with rare diseases, speech disorders, and mental disorders. Furthermore, compared to SP, VSP is more standardized because educators control its design, programming, delivery, and use. Reger used VSP to help healthcare workers improve their motivational interviewing skills. Guetterman proposed that VSP can improve medical students' empathic communication abilities. Du assessed the history-taking skills of nursing students in China using VSP and found that VSP was effective in achieving relatively objective, standardized, and consistent education evaluation."

2b) In INTRODUCTION: Specific objectives or hypotheses

Does your paper address CONSORT subitem 2b? *

Copy and paste relevant sections from the manuscript (include quotes in quotation marks "like this" to indicate direct quotes from your manuscript), or elaborate on this item by providing additional information not in the ms, or briefly explain why the item is not applicable/relevant for your study

Yes. "In this study, we compared the effect of VSP-TCM and traditional academic training on students' clinical competence. We hypothesized that medical students who received VSP-TCM training would perform better than those who received traditional academic training in the following areas: 1) medical history collection; 2) medical writing; 3) syndrome differentiation and treatment; and 4) self-study ability. Finally, we hypothesized that students who received VSP-TCM training would be more satisfied than those who received traditional academic training."

METHODS

3a) Description of trial design (such as parallel, factorial) including allocation ratio

Does your paper address CONSORT subitem 3a? *

Copy and paste relevant sections from the manuscript (include quotes in quotation marks "like this" to indicate direct quotes from your manuscript), or elaborate on this item by providing additional information not in the ms, or briefly explain why the item is not applicable/relevant for your study

Yes. "First, we conducted a questionnaire investigation to gain the perspectives of TCM students who have taken the PTC-IMTCM course. The final version of the VSP-TCM system was designed and developed based on the characteristics of TCM and the results of the questionnaire investigation. Subsequently, we conducted a single-blind, two-group, parallel-training randomized trial to compare the effectiveness of VSP-TCM and academic training for improving clinical competence among TCM medical students. Figure 1 shows the flow of this study."

3b) Important changes to methods after trial commencement (such as eligibility criteria), with reasons

Does your paper address CONSORT subitem 3b? *

Copy and paste relevant sections from the manuscript (include quotes in quotation marks "like this" to indicate direct quotes from your manuscript), or elaborate on this item by providing additional information not in the ms, or briefly explain why the item is not applicable/relevant for your study

No. The study was conducted in strict accordance with the study protocol. There was no change.

3b-i) Bug fixes, Downtimes, Content Changes

Bug fixes, Downtimes, Content Changes: ehealth systems are often dynamic systems. A description of changes to methods therefore also includes important changes made on the intervention or comparator during the trial (e.g., major bug fixes or changes in the functionality or content) (5-iii) and other “unexpected events” that may have influenced study design such as staff changes, system failures/downtimes, etc. [2].

subitem not at all important

1 ○

2 ○

3 ◉

4 ○

5 ○

essential

清除所选内容

Does your paper address subitem 3b-i?

Copy and paste relevant sections from the manuscript (include quotes in quotation marks "like this" to indicate direct quotes from your manuscript), or elaborate on this item by providing additional information not in the ms, or briefly explain why the item is not applicable/relevant for your study

您的回答

4a) Eligibility criteria for participants

Does your paper address CONSORT subitem 4a? *

Copy and paste relevant sections from the manuscript (include quotes in quotation marks "like this" to indicate direct quotes from your manuscript), or elaborate on this item by providing additional information not in the ms, or briefly explain why the item is not applicable/relevant for your study

Yes. "TCM (5+3 integration) students in their second year at CDUTCM were included. The exclusion criteria were as follows: 1) participants who have received VSP or SP training; 2) have taken courses related to IMTCM, such as PTC-IMTCM and reception and clinical thinking skills; and 3) failed to adhere to the study schedule or withdrew from the study."

4a-i) Computer / Internet literacy

Computer / Internet literacy is often an implicit “de facto” eligibility criterion - this should be explicitly clarified.

subitem not at all important

1 ○

2 ○

3 ○

4 ○

5 ◉

essential

清除所选内容

Does your paper address subitem 4a-i?

Copy and paste relevant sections from the manuscript (include quotes in quotation marks "like this" to indicate direct quotes from your manuscript), or elaborate on this item by providing additional information not in the ms, or briefly explain why the item is not applicable/relevant for your study

您的回答

4a-ii) Open vs. closed, web-based vs. face-to-face assessments:

Open vs. closed, web-based vs. face-to-face assessments: Mention how participants were recruited (online vs. offline), e.g., from an open access website or from a clinic, and clarify if this was a purely web-based trial, or there were face-to-face components (as part of the intervention or for assessment), i.e., to what degree got the study team to know the participant. In online-only trials, clarify if participants were quasi-anonymous and whether having multiple identities was possible or whether technical or logistical measures (e.g., cookies, email confirmation, phone calls) were used to detect/prevent these.

subitem not at all important

1 ○

2 ○

3 ○

4 ○

5 ◉

essential

清除所选内容

Does your paper address subitem 4a-ii? *

Copy and paste relevant sections from the manuscript (include quotes in quotation marks "like this" to indicate direct quotes from your manuscript), or elaborate on this item by providing additional information not in the ms, or briefly explain why the item is not applicable/relevant for your study

Yes. "The participants were recruited offline from 112 TCM (5+3 integration) sophomores enrolled in CDUTCM in 2019. All participants have provided written informed consent before participating in the study. Based on previous studies, a minimum sample size of 78 is required."

4a-iii) Information giving during recruitment

Information given during recruitment. Specify how participants were briefed for recruitment and in the informed consent procedures (e.g., publish the informed consent documentation as appendix, see also item X26), as this information may have an effect on user self-selection, user expectation and may also bias results.

subitem not at all important

1 ○

2 ○

3 ○

4 ○

5 ◉

essential

清除所选内容

Does your paper address subitem 4a-iii?

Copy and paste relevant sections from the manuscript (include quotes in quotation marks "like this" to indicate direct quotes from your manuscript), or elaborate on this item by providing additional information not in the ms, or briefly explain why the item is not applicable/relevant for your study

您的回答

4b) Settings and locations where the data were collected

Does your paper address CONSORT subitem 4b? *

Copy and paste relevant sections from the manuscript (include quotes in quotation marks "like this" to indicate direct quotes from your manuscript), or elaborate on this item by providing additional information not in the ms, or briefly explain why the item is not applicable/relevant for your study

Yes. "This study was initiated on February 26, 2020, and concluded on August 20, 2021. From the start of the study to March 2021, staff not involved in the study conducted a pre-questionnaire investigation and gathered data on the participants' performance in basic TCM and WM courses. Then, the 13-week PTC-IMTCM course was conducted from April 1, 2021, to July 10, 2021 (first 12 weeks, training; 13th week, evaluation). Finally, investigators who did not take part in the study collected and analyzed the data by August 20, 2021."

4b-i) Report if outcomes were (self-)assessed through online questionnaires

Clearly report if outcomes were (self-)assessed through online questionnaires (as common in web-based trials) or otherwise.

subitem not at all important

1 ○

2 ○

3 ○

4 ○

5 ◉

essential

清除所选内容

Does your paper address subitem 4b-i? *

Copy and paste relevant sections from the manuscript (include quotes in quotation marks "like this" to indicate direct quotes from your manuscript), or elaborate on this item by providing additional information not in the ms, or briefly explain why the item is not applicable/relevant for your study

Yes. "A previous study's questionnaire was modified for a new audience [8]. After the course, a survey was administered to both groups to assess the students' attitudes toward the course, command of knowledge, and proficiency in clinical skills to help us optimize the course. In addition, we conducted a questionnaire investigation among 15 teachers responsible for PTC-IMTCM to assess the potential impact of VSP-TCM on their work."

4b-ii) Report how institutional affiliations are displayed

Report how institutional affiliations are displayed to potential participants [on ehealth media], as affiliations with prestigious hospitals or universities may affect volunteer rates, use, and reactions with regards to an intervention.(Not a required item – describe only if this may bias results)

subitem not at all important

- 1 ○
- 2 ◉
- 3 ○
- 4 ○
- 5 ○

essential

清除所选内容

Does your paper address subitem 4b-ii?

Copy and paste relevant sections from the manuscript (include quotes in quotation marks "like this" to indicate direct quotes from your manuscript), or elaborate on this item by providing additional information not in the ms, or briefly explain why the item is not applicable/relevant for your study

您的回答

5) The interventions for each group with sufficient details to allow replication, including how and when they were actually administered

5-i) Mention names, credential, affiliations of the developers, sponsors, and owners

Mention names, credential, affiliations of the developers, sponsors, and owners [6] (if authors/evaluators are owners or developer of the software, this needs to be declared in a “Conflict of interest” section or mentioned elsewhere in the manuscript).

subitem not at all important

1 ○

2 ○

3 ◉

4 ○

5 ○

essential

清除所选内容

Does your paper address subitem 5-i?

Copy and paste relevant sections from the manuscript (include quotes in quotation marks "like this" to indicate direct quotes from your manuscript), or elaborate on this item by providing additional information not in the ms, or briefly explain why the item is not applicable/relevant for your study

您的回答

5-ii) Describe the history/development process

Describe the history/development process of the application and previous formative evaluations (e.g., focus groups, usability testing), as these will have an impact on adoption/use rates and help with interpreting results.

subitem not at all important

1 ○

2 ○

3 ◉

4 ○

5 ○

essential

清除所选内容

Does your paper address subitem 5-ii?

Copy and paste relevant sections from the manuscript (include quotes in quotation marks "like this" to indicate direct quotes from your manuscript), or elaborate on this item by providing additional information not in the ms, or briefly explain why the item is not applicable/relevant for your study

您的回答

5-iii) Revisions and updating

Revisions and updating. Clearly mention the date and/or version number of the application/intervention (and comparator, if applicable) evaluated, or describe whether the intervention underwent major changes during the evaluation process, or whether the development and/or content was “frozen” during the trial. Describe dynamic components such as news feeds or changing content which may have an impact on the replicability of the intervention (for unexpected events see item 3b).

subitem not at all important

1 ○

2 ○

3 ◉

4 ○

5 ○

essential

清除所选内容

Does your paper address subitem 5-iii?

Copy and paste relevant sections from the manuscript (include quotes in quotation marks "like this" to indicate direct quotes from your manuscript), or elaborate on this item by providing additional information not in the ms, or briefly explain why the item is not applicable/relevant for your study

您的回答

5-iv) Quality assurance methods

Provide information on quality assurance methods to ensure accuracy and quality of information provided [1], if applicable.

subitem not at all important

1 ○

2 ○

3 ○

4 ◉

5 ○

essential

清除所选内容

Does your paper address subitem 5-iv?

Copy and paste relevant sections from the manuscript (include quotes in quotation marks "like this" to indicate direct quotes from your manuscript), or elaborate on this item by providing additional information not in the ms, or briefly explain why the item is not applicable/relevant for your study

您的回答

5-v) Ensure replicability by publishing the source code, and/or providing screenshots/screen-capture video, and/or providing flowcharts of the algorithms used

Ensure replicability by publishing the source code, and/or providing screenshots/screen-capture video, and/or providing flowcharts of the algorithms used. Replicability (i.e., other researchers should in principle be able to replicate the study) is a hallmark of scientific reporting.

subitem not at all important

1 ○

2 ○

3 ◉

4 ○

5 ○

essential

清除所选内容

Does your paper address subitem 5-v?

Copy and paste relevant sections from the manuscript (include quotes in quotation marks "like this" to indicate direct quotes from your manuscript), or elaborate on this item by providing additional information not in the ms, or briefly explain why the item is not applicable/relevant for your study

您的回答

5-vi) Digital preservation

Digital preservation: Provide the URL of the application, but as the intervention is likely to change or disappear over the course of the years; also make sure the intervention is archived (Internet Archive, webcitation.org, and/or publishing the source code or screenshots/videos alongside the article). As pages behind login screens cannot be archived, consider creating demo pages which are accessible without login.

subitem not at all important

1 ○

2 ○

3 ○

4 ◉

5 ○

essential

清除所选内容

Does your paper address subitem 5-vi?

Copy and paste relevant sections from the manuscript (include quotes in quotation marks "like this" to indicate direct quotes from your manuscript), or elaborate on this item by providing additional information not in the ms, or briefly explain why the item is not applicable/relevant for your study

您的回答

5-vii) Access

Access: Describe how participants accessed the application, in what setting/context, if they had to pay (or were paid) or not, whether they had to be a member of specific group. If known, describe how participants obtained “access to the platform and Internet” [1]. To ensure access for editors/reviewers/readers, consider to provide a “backdoor” login account or demo mode for reviewers/readers to explore the application (also important for archiving purposes, see vi).

subitem not at all important

1 ○

2 ○

3 ◉

4 ○

5 ○

essential

清除所选内容

Does your paper address subitem 5-vii? *

Copy and paste relevant sections from the manuscript (include quotes in quotation marks "like this" to indicate direct quotes from your manuscript), or elaborate on this item by providing additional information not in the ms, or briefly explain why the item is not applicable/relevant for your study

Yes. "URL: http://chm.doctor-u.cn/cdutcm/clinic/login.html"

5-viii) Mode of delivery, features/functionalities/components of the intervention and comparator, and the theoretical framework

Describe mode of delivery, features/functionalities/components of the intervention and comparator, and the theoretical framework [6] used to design them (instructional strategy [1], behaviour change techniques, persuasive features, etc., see e.g., [7, 8] for terminology). This includes an in-depth description of the content (including where it is coming from and who developed it) [1],” whether [and how] it is tailored to individual circumstances and allows users to track their progress and receive feedback” [6]. This also includes a description of communication delivery channels and – if computer-mediated communication is a component – whether communication was synchronous or asynchronous [6]. It also includes information on presentation strategies [1], including page design principles, average amount of text on pages, presence of hyperlinks to other resources, etc. [1].

subitem not at all important

1 ○

2 ○

3 ○

4 ◉

5 ○

essential

清除所选内容

Does your paper address subitem 5-viii? *

Copy and paste relevant sections from the manuscript (include quotes in quotation marks "like this" to indicate direct quotes from your manuscript), or elaborate on this item by providing additional information not in the ms, or briefly explain why the item is not applicable/relevant for your study

Yes. "The operation procedures of VSP-TCM (Figure 2) were as follows: 1) after loging in, select a disease to practice; 2) send commands via voice or text to obtain medical history, including chief complaints, current medical history, past medical history, personal history, and family history; 3) perform targeted physical examinations according to collected medical history, including vital signs, various systems, and TCM tongue and pulse; 4) conduct appropriate auxiliary examinations, including laboratory and imaging examinations; 5) perform diagnosis according to the above information, including diagnosis and antidiastole of TCM and Western medicine (WM) and TCM syndrome differentiation; 6) create treatment plans based on specific diagnoses, including TCM and WM treatment; 7) inform precautions to the patient; and 8) end the medical visit. The system then summarizes the users' knowledge of the disease and evaluates their performance in real-time."

5-ix) Describe use parameters

Describe use parameters (e.g., intended "doses" and optimal timing for use). Clarify what instructions or recommendations were given to the user, e.g., regarding timing, frequency, heaviness of use, if any, or was the intervention used ad libitum.

subitem not at all important

1 ○

2 ○

3 ○

4 ◉

5 ○

essential

清除所选内容

Does your paper address subitem 5-ix?

Copy and paste relevant sections from the manuscript (include quotes in quotation marks "like this" to indicate direct quotes from your manuscript), or elaborate on this item by providing additional information not in the ms, or briefly explain why the item is not applicable/relevant for your study

您的回答

5-x) Clarify the level of human involvement

Clarify the level of human involvement (care providers or health professionals, also technical assistance) in the e-intervention or as co-intervention (detail number and expertise of professionals involved, if any, as well as “type of assistance offered, the timing and frequency of the support, how it is initiated, and the medium by which the assistance is delivered”. It may be necessary to distinguish between the level of human involvement required for the trial, and the level of human involvement required for a routine application outside of a RCT setting (discuss under item 21 – generalizability).

subitem not at all important

- 1 ○
- 2 ○
- 3 ◉
- 4 ○
- 5 ○

essential

清除所选内容

Does your paper address subitem 5-x?

Copy and paste relevant sections from the manuscript (include quotes in quotation marks "like this" to indicate direct quotes from your manuscript), or elaborate on this item by providing additional information not in the ms, or briefly explain why the item is not applicable/relevant for your study

您的回答

5-xi) Report any prompts/reminders used

Report any prompts/reminders used: Clarify if there were prompts (letters, emails, phone calls, SMS) to use the application, what triggered them, frequency etc. It may be necessary to distinguish between the level of prompts/reminders required for the trial, and the level of prompts/reminders for a routine application outside of a RCT setting (discuss under item 21 – generalizability).

subitem not at all important

- 1 (selected)
- 2
- 3
- 4
- 5

essential

清除所选内容

Does your paper address subitem 5-xi? *

Copy and paste relevant sections from the manuscript (include quotes in quotation marks "like this" to indicate direct quotes from your manuscript), or elaborate on this item by providing additional information not in the ms, or briefly explain why the item is not applicable/relevant for your study

No. At present, VSP-TCM is mainly carried out in the classroom. After class, students can volunteer for training. The system does not prompt information.

5-xii) Describe any co-interventions (incl. training/support)

Describe any co-interventions (incl. training/support): Clearly state any interventions that are provided in addition to the targeted eHealth intervention, as ehealth intervention may not be designed as stand-alone intervention. This includes training sessions and support [1]. It may be necessary to distinguish between the level of training required for the trial, and the level of training for a routine application outside of a RCT setting (discuss under item 21 – generalizability.

subitem not at all important

1 ○

2 ○

3 ○

4 ○

5 ◉

essential

清除所选内容

Does your paper address subitem 5-xii? *

Copy and paste relevant sections from the manuscript (include quotes in quotation marks "like this" to indicate direct quotes from your manuscript), or elaborate on this item by providing additional information not in the ms, or briefly explain why the item is not applicable/relevant for your study

Yes. "PTC-IMTCM was conducted in the second semester of sophomore year (36 class hours, 3 class hours/week, a total of 12 weeks). Both groups had the same teaching materials, such as PowerPoint presentations and textbooks. The VSP-TCM group received 36 class hours of VSP-TCM training. The teaching methods were as follows: 1) teacher discussed the fundamentals of the disease, including etiology, pathogenesis, diagnosis, antidiastole, syndrome differentiation and treatment, and key points of history collection; 2) students logged into the VSP-TCM system, selected a disease to practice, and entered the simulated medical scene; 3) students completed the diagnosis and treatment with VSP following the process shown in Figure 2 within the specified time frame; 4) students repeated the exercise according to feedback from the VSP-TCM system; and 5) students voluntarily completed after-school exercises and recorded their learning time. The control group received 36 class hours of academic training, which included teacher teaching and group discussion. Concretely, the teaching methods were as follows: 1) teacher discussed the fundamentals of the disease and syndrome, similar to the VSP-TCM group; 2) students were randomly assigned to several study groups; 3) students discussed the case provided by the teacher and made a diagnosis and suggested treatments for the disease within the specified time frame; 4) the teacher evaluated the answers and provided feedback to the students; and 5) students voluntarily completed after-school exercises and recorded their learning time."

6a) Completely defined pre-specified primary and secondary outcome measures, including how and when they were assessed

Does your paper address CONSORT subitem 6a? *

Copy and paste relevant sections from the manuscript (include quotes in quotation marks "like this" to indicate direct quotes from your manuscript), or elaborate on this item by providing additional information not in the ms, or briefly explain why the item is not applicable/relevant for your study

Yes. "Evaluation of Training Effectiveness
Formative Evaluation
Mini Clinical Evaluation Exercise (Mini-CEX), a tool developed by the American College of Internal Medicine in 1995 based on the traditional clinical evaluation exercise, has teaching functions and was used to assess the residents' clinical ability. Mini-CEX has several advantages, including direct observation, simple form, a key evaluation, and real-time feedback. According to a 9-point evaluation system, the scale comprehensively evaluates the clinical competence of residents from four aspects: history collection ability, physical examination ability, clinical judgment ability, and overall ability. To better evaluate the students' clinical ability, we slightly modified the Mini-CEX according to Chen's research. The modified Mini-CEX could evaluate clinical competence through five aspects: medical interview ability, physical examination ability, clinical judgment ability, disease treatment ability, and comprehensive ability. Multimedia Appendix 1 demonstrates the modified Mini-CEX and its usage for formative evaluation.
Summative Assessment
After the course, the students were given 1 week to review for the assessment, and the summative assessment was conducted on a day in week 13. The summative assessments were divided into online and offline assessments. The participants completed the systematic knowledge test of PTC-IMTCM online before entering the clinical skills center of CDUTCM for offline assessment. The offline assessment included the following steps: 1) collecting medical history from OSP; 2) medical writing; and 3) syndrome differentiation and treatment.
Online Systematic Knowledge Test
After the course, the participants completed an objective and standardized online case-based examination. The students had 90 minutes to complete six cases. For the first five cases, there were five multiple-choice questions in each case, and students were required to select the right diagnosis, syndrome, treatment principle, treatment method, and prescription. Each multiple-choice question was worth 2 points. The sixth case was a case analysis question. Students were required to provide TCM diagnosis (5 points), syndrome differentiation (5 points), syndrome differentiation basis (15 points), treatment (5 points), prescription (5 points), and prescription analysis (15 points) of the patient in the case. The examination had a total score of 100.
Offline Clinical Skill Test
Following the online examination, the students proceeded to the clinical skills center of CDUTCM for a 45-minute offline clinical skills test. For this test, we used previously developed offline clinical skills assessment methods [8]. Students encountered OSP that we had previously trained during testing at the site. Students had 15 minutes to complete the medical history collection process, including a medical interview and physical examination. They were then given 30 minutes to complete medical records and treatment based on syndrome differentiation.
Scores for the application of TCM technology
In the assessment process, a TCM professional who was not part of the research team and did not participate in activities such as teaching and proposition scored the students' clinical skills using a pre-established checklist. The checklist included the following items: introduction (4 points), chief complaint (8 points), current medical history (30 points), past medical history (12 points), personal medical history (12 points), family medical history (8 points), physical examination and four TCM examinations (16 points), and summary (10 points).
Scores of written medical records
The medical writing checklist included the following items: general information (3 points) and chief complaint (5 points), current medical history (30 points), past medical history (10 points), personal medical history (10 points), family medical history (6 points), physical

[illegible]
examination (20 points), and four TCM examinations (16 points). Individual item scores were calculated and processed.
Scores of TCM syndrome differentiation and therapeutic regimen
The following were the content and scores of TCM treatment based on syndrome differentiation: TCM diagnosis (6 points); basis of TCM diagnosis (6 points); WM diagnosis (6 points); basis of WM diagnosis (14 points); TCM syndrome type (10 points); analysis of TCM syndrome differentiation (24 points); TCM treatment method (8 points); formula (8 points); medication, administration method, and corresponding dosage (14 points); and medical advice (4 points). The examination was scored using predetermined standards.
OSP real-time assessment scores
Following each interaction, OSP used the Arizona Clinical Interviewing Rating (ACIR) to assess students' interpersonal communication and interview skills. The ACIR was a 20-item scale, with points ranging from 1–5 points, with 5 being the highest.
Post-course Feedback Questionnaire
A previous study's questionnaire was modified for a new audience. After the course, a survey was administered to both groups to assess the students' attitudes toward the course, command of knowledge, and proficiency in clinical skills to help us optimize the course. In addition, we conducted a questionnaire investigation among 15 teachers responsible for PTC-IMTCM to assess the potential impact of VSP-TCM on their work."

6a-i) Online questionnaires: describe if they were validated for online use and apply CHERRIES items to describe how the questionnaires were designed/deployed

If outcomes were obtained through online questionnaires, describe if they were validated for online use and apply CHERRIES items to describe how the questionnaires were designed/deployed [9].

subitem not at all important

1 ○

2 ○

3 ○

4 ◉

5 ○

essential

清除所选内容

Does your paper address subitem 6a-i?

Copy and paste relevant sections from manuscript text

您的回答

6a-ii) Describe whether and how “use” (including intensity of use/dosage) was defined/measured/monitored

Describe whether and how “use” (including intensity of use/dosage) was defined/measured/monitored (logins, logfile analysis, etc.). Use/adoption metrics are important process outcomes that should be reported in any ehealth trial.

subitem not at all important

1 ○

2 ◉

3 ○

4 ○

5 ○

essential

清除所选内容

Does your paper address subitem 6a-ii?

Copy and paste relevant sections from manuscript text

您的回答

6a-iii) Describe whether, how, and when qualitative feedback from participants was obtained

Describe whether, how, and when qualitative feedback from participants was obtained (e.g., through emails, feedback forms, interviews, focus groups).

subitem not at all important

1 ○

2 ○

3 ○

4 ◉

5 ○

essential

清除所选内容

Does your paper address subitem 6a-iii?

Copy and paste relevant sections from manuscript text

Yes. "A previous study’s questionnaire was modified for a new audience [8]. After the course, a survey was administered to both groups to assess the students’ attitudes toward the course, command of knowledge, and proficiency in clinical skills to help us optimize the course. In addition, we conducted a questionnaire investigation among 15 teachers responsible for PTC-IMTCM to assess the potential impact of VSP-TCM on their work."

6b) Any changes to trial outcomes after the trial commenced, with reasons

Does your paper address CONSORT subitem 6b? *

Copy and paste relevant sections from the manuscript (include quotes in quotation marks "like this" to indicate direct quotes from your manuscript), or elaborate on this item by providing additional information not in the ms, or briefly explain why the item is not applicable/relevant for your study

No. The study was conducted in strict accordance with the study protocol. There was no change to trial outcomes.

7a) How sample size was determined

NPT: When applicable, details of whether and how the clustering by care provides or centers was addressed

7a-i) Describe whether and how expected attrition was taken into account when calculating the sample size

Describe whether and how expected attrition was taken into account when calculating the sample size.

subitem not at all important

1 ○

2 ○

3 ○

4 ◉

5 ○

essential

清除所选内容

Does your paper address subitem 7a-i?

Copy and paste relevant sections from manuscript title (include quotes in quotation marks "like this" to indicate direct quotes from your manuscript), or elaborate on this item by providing additional information not in the ms, or briefly explain why the item is not applicable/relevant for your study

您的回答

7b) When applicable, explanation of any interim analyses and stopping guidelines

Does your paper address CONSORT subitem 7b? *

Copy and paste relevant sections from the manuscript (include quotes in quotation marks "like this" to indicate direct quotes from your manuscript), or elaborate on this item by providing additional information not in the ms, or briefly explain why the item is not applicable/relevant for your study

Yes. "The exclusion criteria were as follows: 1) participants who have received VSP or SP training; 2) have taken courses related to IMTCM, such as PTC-IMTCM and reception and clinical thinking skills; and 3) failed to adhere to the study schedule or withdrew from the study."

8a) Method used to generate the random allocation sequence

NPT: When applicable, how care providers were allocated to each trial group

Does your paper address CONSORT subitem 8a? *

Copy and paste relevant sections from the manuscript (include quotes in quotation marks "like this" to indicate direct quotes from your manuscript), or elaborate on this item by providing additional information not in the ms, or briefly explain why the item is not applicable/relevant for your study

Yes. "Using computer-generated randomization, the participants were randomly assigned to either the VSP-TCM group or the control group in a 1:1 ratio. The randomization was performed by individuals who were not in contact with the participants. The participants, staff, and investigators were blinded to the training assignments. Data analysis was deferred until all data were collected by investigators who were blinded to the outcomes."

8b) Type of randomisation; details of any restriction (such as blocking and block size)

Does your paper address CONSORT subitem 8b? *

Copy and paste relevant sections from the manuscript (include quotes in quotation marks "like this" to indicate direct quotes from your manuscript), or elaborate on this item by providing additional information not in the ms, or briefly explain why the item is not applicable/relevant for your study

Yes. "Using computer-generated randomization, the participants were randomly assigned to either the VSP-TCM group or the control group in a 1:1 ratio. The randomization was performed by individuals who were not in contact with the participants. The participants, staff, and investigators were blinded to the training assignments. Data analysis was deferred until all data were collected by investigators who were blinded to the outcomes."

9) Mechanism used to implement the random allocation sequence (such as sequentially numbered containers), describing any steps taken to conceal the sequence until interventions were assigned

Does your paper address CONSORT subitem 9? *

Copy and paste relevant sections from the manuscript (include quotes in quotation marks "like this" to indicate direct quotes from your manuscript), or elaborate on this item by providing additional information not in the ms, or briefly explain why the item is not applicable/relevant for your study

Yes. "Using computer-generated randomization, the participants were randomly assigned to either the VSP-TCM group or the control group in a 1:1 ratio. The randomization was performed by individuals who were not in contact with the participants. The participants, staff, and investigators were blinded to the training assignments. Data analysis was deferred until all data were collected by investigators who were blinded to the outcomes."

10) Who generated the random allocation sequence, who enrolled participants, and who assigned participants to interventions

Does your paper address CONSORT subitem 10? *

Copy and paste relevant sections from the manuscript (include quotes in quotation marks "like this" to indicate direct quotes from your manuscript), or elaborate on this item by providing additional information not in the ms, or briefly explain why the item is not applicable/relevant for your study

Yes. "Using computer-generated randomization, the participants were randomly assigned to either the VSP-TCM group or the control group in a 1:1 ratio. The randomization was performed by individuals who were not in contact with the participants. The participants, staff, and investigators were blinded to the training assignments. Data analysis was deferred until all data were collected by investigators who were blinded to the outcomes."

11a) If done, who was blinded after assignment to interventions (for example, participants, care providers, those assessing outcomes) and how

NPT: Whether or not administering co-interventions were blinded to group assignment

11a-i) Specify who was blinded, and who wasn't

Specify who was blinded, and who wasn't. Usually, in web-based trials it is not possible to blind the participants [1, 3] (this should be clearly acknowledged), but it may be possible to blind outcome assessors, those doing data analysis or those administering co-interventions (if any).

subitem not at all important

- 1 ○
- 2 ○
- 3 ○
- 4 ◉
- 5 ○

essential

清除所选内容

Does your paper address subitem 11a-i? *

Copy and paste relevant sections from the manuscript (include quotes in quotation marks "like this" to indicate direct quotes from your manuscript), or elaborate on this item by providing additional information not in the ms, or briefly explain why the item is not applicable/relevant for your study

Yes. "Using computer-generated randomization, the participants were randomly assigned to either the VSP-TCM group or the control group in a 1:1 ratio. The randomization was performed by individuals who were not in contact with the participants. The participants, staff, and investigators were blinded to the training assignments. Data analysis was deferred until all data were collected by investigators who were blinded to the outcomes."

11a-ii) Discuss e.g., whether participants knew which intervention was the “intervention of interest” and which one was the “comparator”

Informed consent procedures (4a-ii) can create biases and certain expectations - discuss e.g., whether participants knew which intervention was the “intervention of interest” and which one was the “comparator”.

subitem not at all important

1 ○

2 ○

3 ◉

4 ○

5 ○

essential

清除所选内容

Does your paper address subitem 11a-ii?

Copy and paste relevant sections from the manuscript (include quotes in quotation marks "like this" to indicate direct quotes from your manuscript), or elaborate on this item by providing additional information not in the ms, or briefly explain why the item is not applicable/relevant for your study

您的回答

11b) If relevant, description of the similarity of interventions
(this item is usually not relevant for ehealth trials as it refers to similarity of a placebo or sham intervention to a active medication/intervention)

Does your paper address CONSORT subitem 11b? *

Copy and paste relevant sections from the manuscript (include quotes in quotation marks "like this" to indicate direct quotes from your manuscript), or elaborate on this item by providing additional information not in the ms, or briefly explain why the item is not applicable/relevant for your study

Yes. "Teachers in the course group underwent standardized training 1 month before the course started. The standardized training planning was set based on the “Eight-Year Undergraduate Talent Training Guide of Traditional Chinese Medicine” issued by CDUTCM. Subsequently, experienced clinical experts were invited to assess the teachers’ qualifications. Finally, we selected two teachers who were closely matched in terms of age, sex, teaching experience, and teaching style. Using a random coin toss, they were then assigned to either the VSP-TCM group or the control group."

12a) Statistical methods used to compare groups for primary and secondary outcomes
NPT: When applicable, details of whether and how the clustering by care providers or centers was addressed

Does your paper address CONSORT subitem 12a? *

Copy and paste relevant sections from the manuscript (include quotes in quotation marks "like this" to indicate direct quotes from your manuscript), or elaborate on this item by providing additional information not in the ms, or briefly explain why the item is not applicable/relevant for your study

Yes. "A blinded research analyst conducted the test. The intraclass correlation coefficient (ICC) was calculated to determine the consistency of the Mini-CEX scores. The statistical analysis was conducted using SPSS 25.0 (IBM, Armonk, NY). Continuous variables were expressed as mean ± standard deviation (SD) and categorical variables as frequency or percentage. The Kolmogorov–Smirnov test was used to determine the normality of all the data. When the data has a normal distribution, the independent-samples t-test was used, otherwise, the Mann–Whitney test was employed. The Chi-squared ($\chi 2$) test was used to compare proportions. P-values ≤0.05 indicated significant differences."

12a-i) Imputation techniques to deal with attrition / missing values

Imputation techniques to deal with attrition / missing values: Not all participants will use the intervention/comparator as intended and attrition is typically high in ehealth trials. Specify how participants who did not use the application or dropped out from the trial were treated in the statistical analysis (a complete case analysis is strongly discouraged, and simple imputation techniques such as LOCF may also be problematic [4]).

subitem not at all important

1 ○

2 ○

3 ◉

4 ○

5 ○

essential

清除所选内容

Does your paper address subitem 12a-i? *

Copy and paste relevant sections from the manuscript (include quotes in quotation marks "like this" to indicate direct quotes from your manuscript), or elaborate on this item by providing additional information not in the ms, or briefly explain why the item is not applicable/relevant for your study

Yes. "TCM (5+3 integration) students in their second year at CDUTCM were included. The exclusion criteria were as follows: 1) participants who have received VSP or SP training; 2) have taken courses related to IMTCM, such as PTC-IMTCM and reception and clinical thinking skills; and 3) failed to adhere to the study schedule or withdrew from the study."

12b) Methods for additional analyses, such as subgroup analyses and adjusted analyses

Does your paper address CONSORT subitem 12b? *

Copy and paste relevant sections from the manuscript (include quotes in quotation marks "like this" to indicate direct quotes from your manuscript), or elaborate on this item by providing additional information not in the ms, or briefly explain why the item is not applicable/relevant for your study

No. There were no subgroup or adjust analysis in this study.

X26) REB/IRB Approval and Ethical Considerations [recommended as subheading under "Methods"] (not a CONSORT item)

X26-i) Comment on ethics committee approval

subitem not at all important

1 ○

2 ○

3 ○

4 ○

5 ◉

essential

清除所选内容

Does your paper address subitem X26-i?

Copy and paste relevant sections from the manuscript (include quotes in quotation marks "like this" to indicate direct quotes from your manuscript), or elaborate on this item by providing additional information not in the ms, or briefly explain why the item is not applicable/relevant for your study

Yes. "The study was approved by the Ethics Committee of CDUTCM (approval no. 25382) and adhered to the principles of the Declaration of Helsinki. The curriculum planning adhered to the guidelines set by the "Undergraduate Medical Education Standards - Traditional Chinese Medicine" issued by the National Advisory Committee on Higher Traditional Chinese Medicine Education of the Ministry of Education and the "Eight-Year Undergraduate Talent Training Guide of Traditional Chinese Medicine" issued by CDUTCM. The current study followed the CONSORT-EHEALTH (Consolidated Standards of Reporting Trials of Electronic and Mobile HEalth Applications and onLine TeleHealth) checklist to report its findings [23]. "

x26-ii) Outline informed consent procedures

Outline informed consent procedures e.g., if consent was obtained offline or online (how? Checkbox, etc.?), and what information was provided (see 4a-ii). See [6] for some items to be included in informed consent documents.

subitem not at all important

1 ○

2 ○

3 ○

4 ◉

5 ○

essential

清除所选内容

Does your paper address subitem X26-ii?

Copy and paste relevant sections from the manuscript (include quotes in quotation marks "like this" to indicate direct quotes from your manuscript), or elaborate on this item by providing additional information not in the ms, or briefly explain why the item is not applicable/relevant for your study

您的回答

X26-iii) Safety and security procedures

Safety and security procedures, incl. privacy considerations, and any steps taken to reduce the likelihood or detection of harm (e.g., education and training, availability of a hotline)

subitem not at all important

1 ○

2 ○

3 ◉

4 ○

5 ○

essential

清除所选内容

Does your paper address subitem X26-iii?

Copy and paste relevant sections from the manuscript (include quotes in quotation marks "like this" to indicate direct quotes from your manuscript), or elaborate on this item by providing additional information not in the ms, or briefly explain why the item is not applicable/relevant for your study

您的回答

RESULTS

13a) For each group, the numbers of participants who were randomly assigned, received intended treatment, and were analysed for the primary outcome
NPT: The number of care providers or centers performing the intervention in each group and the number of patients treated by each care provider in each center

Does your paper address CONSORT subitem 13a? *

Copy and paste relevant sections from the manuscript (include quotes in quotation marks "like this" to indicate direct quotes from your manuscript), or elaborate on this item by providing additional information not in the ms, or briefly explain why the item is not applicable/relevant for your study

Yes. "A total of 84 students participated in this study and were randomly assigned to the VSP-TCM group (n=42) and the control group (n=42). There was no significant difference in age (P=.11) and sex (P=.64) between the two groups. At baseline, there was no significant difference between both groups in TCM basic courses (P=.74), WM basic courses (P=.31), or the grade point average (P=.33)"; "Summative Assessment
Online systematic knowledge test
After 12 weeks of the course, participants in the VSP-TCM group mastered the systematic knowledge of the course better than those in the control group (Figure 4A). In the online systematic knowledge test, participants in the VSP-TCM group scored higher than those in the control group (86.62±2.71vs. 85.38±2.62, U=660.5, P=.046).
Offline clinical skills test
Scores for the application of TCM technology
Participants in the VSP-TCM group outperformed those in the control group in receiving VSP-TCM. Participants in the VSP-TCM group performed better in the application of TCM skills (87.86±3.04 vs. 86.19±3.08, t=2.464, P=.02, d=82) (Figure 4B).
Scores of written medical records
VSP-TCM did not provide the expected benefits in improving the participants' ability to write medical records. Participants in the VSP-TCM group scored lower than those in the control group (75.07±3.61 vs. 75.71±2.86, t=0.8945, P=.37, d=82) (Figure 4C).
Scores of TCM syndrome differentiation and therapeutic regimen
VSP-TCM effectively improved the core TCM skills, including syndrome differentiation and treatment. The VSP-TCM group had higher scores for TCM syndrome differentiation and treatment than the control group (90.93±2.42 vs. 89.60±2.86, U=636, P=.03) (Figure 4D).
Real-time assessment scores from OSP
VSP-TCM had a satisfactory effect on improving participants' interpersonal communication and interview skills. The real-time evaluation score from OSP in the VSP-TCM group was significantly higher than the control group (90.67±4.52 vs. 88.24±4.56, U=618.5, P=.02) (Figure 4E)."

13b) For each group, losses and exclusions after randomisation, together with reasons

Does your paper address CONSORT subitem 13b? (NOTE: Preferably, this is shown in a CONSORT flow diagram) *

Copy and paste relevant sections from the manuscript (include quotes in quotation marks "like this" to indicate direct quotes from your manuscript), or elaborate on this item by providing additional information not in the ms, or briefly explain why the item is not applicable/relevant for your study

No. We excluded 28 students from 112 students based on inclusion and exclusion criteria.A total of 84 students were randomized and all completed the study.

13b-i) Attrition diagram

Strongly recommended: An attrition diagram (e.g., proportion of participants still logging in or using the intervention/comparator in each group plotted over time, similar to a survival curve) or other figures or tables demonstrating usage/dose/engagement.

subitem not at all important

- 1 ○
- 2 ○
- 3 ●
- 4 ○
- 5 ○

essential

清除所选内容

Does your paper address subitem 13b-i?

Copy and paste relevant sections from the manuscript or cite the figure number if applicable (include quotes in quotation marks "like this" to indicate direct quotes from your manuscript), or elaborate on this item by providing additional information not in the ms, or briefly explain why the item is not applicable/relevant for your study

您的回答

14a) Dates defining the periods of recruitment and follow-up

Does your paper address CONSORT subitem 14a? *

Copy and paste relevant sections from the manuscript (include quotes in quotation marks "like this" to indicate direct quotes from your manuscript), or elaborate on this item by providing additional information not in the ms, or briefly explain why the item is not applicable/relevant for your study

Yes. "This study was initiated on February 26, 2020, and concluded on August 20, 2021. From the start of the study to March 2021, staff not involved in the study conducted a pre-questionnaire investigation and gathered data on the participants' performance in basic TCM and WM courses. Then, the 13-week PTC-IMTCM course was conducted from April 1, 2021, to July 10, 2021 (first 12 weeks, training; 13th week, evaluation). Finally, investigators who did not take part in the study collected and analyzed the data by August 20, 2021."

14a-i) Indicate if critical "secular events" fell into the study period

Indicate if critical "secular events" fell into the study period, e.g., significant changes in Internet resources available or "changes in computer hardware or Internet delivery resources"

subitem not at all important

1 ○

2 ○

3 ○

4 ○

5 ○

essential

Does your paper address subitem 14a-i?

Copy and paste relevant sections from the manuscript (include quotes in quotation marks "like this" to indicate direct quotes from your manuscript), or elaborate on this item by providing additional information not in the ms, or briefly explain why the item is not applicable/relevant for your study

您的回答

14b) Why the trial ended or was stopped (early)

Does your paper address CONSORT subitem 14b? *

Copy and paste relevant sections from the manuscript (include quotes in quotation marks "like this" to indicate direct quotes from your manuscript), or elaborate on this item by providing additional information not in the ms, or briefly explain why the item is not applicable/relevant for your study

Yes. The trial was conducted in accordance with the study protocol and ended finally.

15) A table showing baseline demographic and clinical characteristics for each group

NPT: When applicable, a description of care providers (case volume, qualification, expertise, etc.) and centers (volume) in each group

Does your paper address CONSORT subitem 15? *

Copy and paste relevant sections from the manuscript (include quotes in quotation marks "like this" to indicate direct quotes from your manuscript), or elaborate on this item by providing additional information not in the ms, or briefly explain why the item is not applicable/relevant for your study

Yes.
Characteristics Training condition P
VSP-TCMa group (n=42) Control group (n=42)
Age(years), mean(SD) 20.69(0.67) 20.85(0.60) .11
Sex，n(%)
Female 28(66.67) 30(71.43) .64
Male 14(33.33) 12(28.57)
Basic courses of TCMb (points), mean(SD) 82.85(6.53) 83.63(6.37) .74
Basic courses of WMc (points), mean(SD) 82.57(3.87) 82.59(4.56) .31
GPAd, mean(SD) 3.27(0.33) 3.24(0.37) .33

15-i) Report demographics associated with digital divide issues

In ehealth trials it is particularly important to report demographics associated with digital divide issues, such as age, education, gender, social-economic status, computer/Internet/ehealth literacy of the participants, if known.

subitem not at all important

1 ○

2 ○

3 ◉

4 ○

5 ○

essential

清除所选内容

Does your paper address subitem 15-i? *

Copy and paste relevant sections from the manuscript (include quotes in quotation marks "like this" to indicate direct quotes from your manuscript), or elaborate on this item by providing additional information not in the ms, or briefly explain why the item is not applicable/relevant for your study

Yes.
Characteristics Training condition P
VSP-TCMa group (n=42) Control group (n=42)
Age(years), mean(SD) 20.69(0.67) 20.85(0.60) .11
Sex，n(%)
Female 28(66.67) 30(71.43) .64
Male 14(33.33) 12(28.57)
Basic courses of TCMb (points), mean(SD) 82.85(6.53) 83.63(6.37) .74
Basic courses of WMc (points), mean(SD) 82.57(3.87) 82.59(4.56) .31
GPAd, mean(SD) 3.27(0.33) 3.24(0.37) .33

16) For each group, number of participants (denominator) included in each analysis and whether the analysis was by original assigned groups

16-i) Report multiple “denominators” and provide definitions

Report multiple “denominators” and provide definitions: Report N’s (and effect sizes) “across a range of study participation [and use] thresholds” [1], e.g., N exposed, N consented, N used more than x times, N used more than y weeks, N participants “used” the intervention/comparator at specific pre-defined time points of interest (in absolute and relative numbers per group). Always clearly define “use” of the intervention.

subitem not at all important

1 ○

2 ○

3 ○

4 ◉

5 ○

essential

清除所选内容

Does your paper address subitem 16-i? *

Copy and paste relevant sections from the manuscript (include quotes in quotation marks "like this" to indicate direct quotes from your manuscript), or elaborate on this item by providing additional information not in the ms, or briefly explain why the item is not applicable/relevant for your study

Yes. A total of 84 students participated in this study and were randomly assigned to the VSP-TCM group (n=42) and the control group (n=42). There was no significant difference in age (P=.11) and sex (P=.64) between the two groups. At baseline, there was no significant difference between both groups in TCM basic courses (P=.74), WM basic courses (P=.31), or the grade point average (P=.33) (Table 2).

16-ii) Primary analysis should be intent-to-treat

Primary analysis should be intent-to-treat, secondary analyses could include comparing only “users”, with the appropriate caveats that this is no longer a randomized sample (see 18-i).

subitem not at all important

1 ○

2 ◉

3 ○

4 ○

5 ○

essential

清除所选内容

Does your paper address subitem 16-ii?

Copy and paste relevant sections from the manuscript (include quotes in quotation marks "like this" to indicate direct quotes from your manuscript), or elaborate on this item by providing additional information not in the ms, or briefly explain why the item is not applicable/relevant for your study

您的回答

17a) For each primary and secondary outcome, results for each group, and the estimated effect size and its precision (such as 95% confidence interval)

Does your paper address CONSORT subitem 17a? *

Copy and paste relevant sections from the manuscript (include quotes in quotation marks "like this" to indicate direct quotes from your manuscript), or elaborate on this item by providing additional information not in the ms, or briefly explain why the item is not applicable/relevant for your study

Yes. "Evaluation of Training Effectiveness
Formative Evaluation
Figure 3 shows the Mini-CEX results for both groups. The consistency among physicians responsible for evaluating the participants' performance was high (ICC=0.82). After 6 weeks of training, participants assigned to the VSP-TCM group gained higher scores in medical interview (7.19±0.63 vs. 6.83±0.81, U=656.5, P=.04), clinical judgment (6.48±0.98 vs. 5.86±1.04, U=590, P=.006), and comprehensive ability (6.71±0.59 vs. 6.40±0.58, U=634.5, P=.02) than those assigned to the control group. However, participants in the VSP-TCM group did not show the expected advantages in physical examination (6.14±1.19 vs. 6.29±1.20, U=830, P=.64) and disease treatment (6.88±0.98 vs. 6.74±1.16, U=827, P=.62), and the score was slightly lower than the control group.
Summative Assessment
Online systematic knowledge test
After 12 weeks of the course, participants in the VSP-TCM group mastered the systematic knowledge of the course better than those in the control group (Figure 4A). In the online systematic knowledge test, participants in the VSP-TCM group scored higher than those in the control group (86.62±2.71vs. 85.38±2.62, U=660.5, P=.046).
Offline clinical skills test
Scores for the application of TCM technology
Participants in the VSP-TCM group outperformed those in the control group in receiving VSP-TCM. Participants in the VSP-TCM group performed better in the application of TCM skills (87.86±3.04 vs. 86.19±3.08, t=2.464, P=.02, d=82) (Figure 4B).
Scores of written medical records
VSP-TCM did not provide the expected benefits in improving the participants' ability to write medical records. Participants in the VSP-TCM group scored lower than those in the control group (75.07±3.61 vs. 75.71±2.86, t=0.8945, P=.37, d=82) (Figure 4C).
Scores of TCM syndrome differentiation and therapeutic regimen
VSP-TCM effectively improved the core TCM skills, including syndrome differentiation and treatment. The VSP-TCM group had higher scores for TCM syndrome differentiation and treatment than the control group (90.93±2.42 vs. 89.60±2.86, U=636, P=.03) (Figure 4D).
Real-time assessment scores from OSP
VSP-TCM had a satisfactory effect on improving participants' interpersonal communication and interview skills. The real-time evaluation score from OSP in the VSP-TCM group was significantly higher than the control group (90.67±4.52 vs. 88.24±4.56, U=618.5, P=.02) (Figure 4E)."

17a-i) Presentation of process outcomes such as metrics of use and intensity of use

In addition to primary/secondary (clinical) outcomes, the presentation of process outcomes such as metrics of use and intensity of use (dose, exposure) and their operational definitions is critical. This does not only refer to metrics of attrition (13-b) (often a binary variable), but also to more continuous exposure metrics such as “average session length”. These must be accompanied by a technical description how a metric like a “session” is defined (e.g., timeout after idle time) [1] (report under item 6a).

subitem not at all important

1 ○

2 ○

3 ◉

4 ○

5 ○

essential

清除所选内容

Does your paper address subitem 17a-i?

Copy and paste relevant sections from the manuscript (include quotes in quotation marks "like this" to indicate direct quotes from your manuscript), or elaborate on this item by providing additional information not in the ms, or briefly explain why the item is not applicable/relevant for your study

您的回答

17b) For binary outcomes, presentation of both absolute and relative effect sizes is recommended

Does your paper address CONSORT subitem 17b? *

Copy and paste relevant sections from the manuscript (include quotes in quotation marks "like this" to indicate direct quotes from your manuscript), or elaborate on this item by providing additional information not in the ms, or briefly explain why the item is not applicable/relevant for your study

Yes. "Table 3 displays the results of the post-course feedback questionnaire administered to the students. Six of the 11 items in the questionnaire showed significant differences between both groups. Of 42 participants in the VSP-TCM group, 39 (93%) believed that the course improved their TCM thinking ability as opposed to 37 participants (88%) in the control group (P=.002). Regarding medical history collection, 38 participants (90%) in the VSP-TCM group found the course beneficial as opposed to 30 participants (72%) in the control group (P=.001). In terms of syndrome differentiation and treatment and critical thinking ability, 38 participants (90%) found VSP-TCM helpful, and 37 participants (88%) found traditional academic training helpful (P=.046). In addition, 40 participants (95%) in the VSP-TCM group gained better clinical comprehensive application ability as opposed to 36 participants (86%) in the control group (P=.009). Furthermore, 36 participants (86%) trained with VSP-TCM grasped better interpersonal communication skills, whereas only 28 students (67%) grasped the same skills in the control group (P=.01). Interestingly, 37 (88%) participants who received VSP-TCM teaching significantly improved their autonomous learning ability; however, only 28 students (67%) endorsed this viewpoint in the control group (P=.01). Overall, participants in both groups were satisfied with the course (P=.23); however, VSP-TCM did not improve their medical writing ability than academic training (P=.13)"; "Table 4 shows the results of the feedback questionnaire administered to teachers. Overall, we found that teachers held a positive perspective toward VSP-TCM. No less than 12 teachers (80%) expressed their belief that VSP-TCM has the potential to become a prominent trend in TCM education and expressed their willingness to integrate it into their teaching practices. Simultaneously, they recognized the benefits of utilizing VSP-TCM to enhance students' motivation for learning (12/15, 80%), regarding it as a valuable adjunct to bedside instruction (15/15, 100%). Furthermore, it is worth mentioning that teachers preferred to develop VSP based on syndromes (14/15, 93%) rather than diseases (11/15, 73%), which highlighted the characteristics of "treatment based on syndrome differentiation." Finally, all teachers unanimously agreed that VSP-TCM has the potential to enhance teaching efficiency while effectively minimizing teaching expenses, thus establishing it as a cost-effective instructional resource."

18) Results of any other analyses performed, including subgroup analyses and adjusted analyses, distinguishing pre-specified from exploratory

Does your paper address CONSORT subitem 18? *

Copy and paste relevant sections from the manuscript (include quotes in quotation marks "like this" to indicate direct quotes from your manuscript), or elaborate on this item by providing additional information not in the ms, or briefly explain why the item is not applicable/relevant for your study

No. There were no subgroup or adjusted analyses in this study.

18-i) Subgroup analysis of comparing only users

A subgroup analysis of comparing only users is not uncommon in ehealth trials, but if done, it must be stressed that this is a self-selected sample and no longer an unbiased sample from a randomized trial (see 16-iii).

subitem not at all important

1 ○

2 ○

3 ◉

4 ○

5 ○

essential

清除所选内容

Does your paper address subitem 18-i?

Copy and paste relevant sections from the manuscript (include quotes in quotation marks "like this" to indicate direct quotes from your manuscript), or elaborate on this item by providing additional information not in the ms, or briefly explain why the item is not applicable/relevant for your study

您的回答

19) All important harms or unintended effects in each group

(for specific guidance see CONSORT for harms)

Does your paper address CONSORT subitem 19? *

Copy and paste relevant sections from the manuscript (include quotes in quotation marks "like this" to indicate direct quotes from your manuscript), or elaborate on this item by providing additional information not in the ms, or briefly explain why the item is not applicable/relevant for your study

No. The VSP-TCM was a virtual clinical and did not cause any harm to the participants.

19-i) Include privacy breaches, technical problems

Include privacy breaches, technical problems. This does not only include physical “harm” to participants, but also incidents such as perceived or real privacy breaches [1], technical problems, and other unexpected/unintended incidents. “Unintended effects” also includes unintended positive effects [2].

subitem not at all important

1 ○

2 ○

3 ◉

4 ○

5 ○

essential

清除所选内容

Does your paper address subitem 19-i?

Copy and paste relevant sections from the manuscript (include quotes in quotation marks "like this" to indicate direct quotes from your manuscript), or elaborate on this item by providing additional information not in the ms, or briefly explain why the item is not applicable/relevant for your study

您的回答

19-ii) Include qualitative feedback from participants or observations from staff/researchers

Include qualitative feedback from participants or observations from staff/researchers, if available, on strengths and shortcomings of the application, especially if they point to unintended/unexpected effects or uses. This includes (if available) reasons for why people did or did not use the application as intended by the developers.

subitem not at all important

1 ○

2 ○

3 ○

4 ◉

5 ○

essential

清除所选内容

Does your paper address subitem 19-ii?

Copy and paste relevant sections from the manuscript (include quotes in quotation marks "like this" to indicate direct quotes from your manuscript), or elaborate on this item by providing additional information not in the ms, or briefly explain why the item is not applicable/relevant for your study

您的回答

DISCUSSION

22) Interpretation consistent with results, balancing benefits and harms, and considering other relevant evidence

NPT: In addition, take into account the choice of the comparator, lack of or partial blinding, and unequal expertise of care providers or centers in each group

22-i) Restate study questions and summarize the answers suggested by the data, starting with primary outcomes and process outcomes (use)

Restate study questions and summarize the answers suggested by the data, starting with primary outcomes and process outcomes (use).

subitem not at all important

1 ○

2 ○

3 ○

4 ○

5 ◉

essential

清除所选内容

Does your paper address subitem 22-i? *

Copy and paste relevant sections from the manuscript (include quotes in quotation marks "like this" to indicate direct quotes from your manuscript), or elaborate on this item by providing additional information not in the ms, or briefly explain why the item is not applicable/relevant for your study

Yes. "This study demonstrated that, compared with academic training, VSP-TCM significantly improved abilities in medical interview, clinical judgment, TCM technology application, and systematic knowledge among TCM students. The VSP-TCM system enabled TCM education to be more efficient and less stressful for teachers. It was also helpful for reforming the course and achieving the goal of training applied talents."

22-ii) Highlight unanswered new questions, suggest future research

Highlight unanswered new questions, suggest future research.

subitem not at all important

1 ○

2 ○

3 ○

4 ◉

5 ○

essential

清除所选内容

Does your paper address subitem 22-ii?

Copy and paste relevant sections from the manuscript (include quotes in quotation marks "like this" to indicate direct quotes from your manuscript), or elaborate on this item by providing additional information not in the ms, or briefly explain why the item is not applicable/relevant for your study

您的回答

20) Trial limitations, addressing sources of potential bias, imprecision, and, if relevant, multiplicity of analyses

20-i) Typical limitations in ehealth trials

Typical limitations in ehealth trials: Participants in ehealth trials are rarely blinded. Ehealth trials often look at a multiplicity of outcomes, increasing risk for a Type I error. Discuss biases due to non-use of the intervention/usability issues, biases through informed consent procedures, unexpected events.

subitem not at all important

1 ○

2 ○

3 ◉

4 ○

5 ○

essential

清除所选内容

Does your paper address subitem 20-i? *

Copy and paste relevant sections from the manuscript (include quotes in quotation marks "like this" to indicate direct quotes from your manuscript), or elaborate on this item by providing additional information not in the ms, or briefly explain why the item is not applicable/relevant for your study

Yes. "Although the findings of this study are positive, it has some limitations that should be acknowledged. First, in this prospective study, we only evaluated the clinical skills of students during and after the course, with no long-term follow-up. We were unaware of how VSP-TCM maintained the trainees' competence. Secondly, VSP-TCM could not convey some physical signs of real patients. For example, TCM-specific pulse conditions can only be displayed through images, preventing trainees from gaining real clinical experience in pulse diagnosis [8]. In addition, it was a single-center study, which affected the universality of the findings. All the participants were from CDUTCM. It was challenging to represent all TCM students. Therefore, future large-scale, multicenter studies are warranted."

21) Generalisability (external validity, applicability) of the trial findings

NPT: External validity of the trial findings according to the intervention, comparators, patients, and care providers or centers involved in the trial

21-i) Generalizability to other populations

Generalizability to other populations: In particular, discuss generalizability to a general Internet population, outside of a RCT setting, and general patient population, including applicability of the study results for other organizations

subitem not at all important

1 ○

2 ○

3 ○

4 ◉

5 ○

essential

清除所选内容

Does your paper address subitem 21-i?

Copy and paste relevant sections from the manuscript (include quotes in quotation marks "like this" to indicate direct quotes from your manuscript), or elaborate on this item by providing additional information not in the ms, or briefly explain why the item is not applicable/relevant for your study

您的回答

21-ii) Discuss if there were elements in the RCT that would be different in a routine application setting

Discuss if there were elements in the RCT that would be different in a routine application setting (e.g., prompts/reminders, more human involvement, training sessions or other co-interventions) and what impact the omission of these elements could have on use, adoption, or outcomes if the intervention is applied outside of a RCT setting.

subitem not at all important

1 ○

2 ○

3 ◉

4 ○

5 ○

essential

清除所选内容

Does your paper address subitem 21-ii?

Copy and paste relevant sections from the manuscript (include quotes in quotation marks "like this" to indicate direct quotes from your manuscript), or elaborate on this item by providing additional information not in the ms, or briefly explain why the item is not applicable/relevant for your study

您的回答

OTHER INFORMATION

23) Registration number and name of trial registry

Does your paper address CONSORT subitem 23? *

Copy and paste relevant sections from the manuscript (include quotes in quotation marks "like this" to indicate direct quotes from your manuscript), or elaborate on this item by providing additional information not in the ms, or briefly explain why the item is not applicable/relevant for your study

No. This was a study investigating the teaching effectiveness of VSP-TCM in TCM education. As this is based on a training course approved by the Ethics Committee of CDUTCM (approval no. 25382), and the training protocol adhered to the "Undergraduate Medical Education Standards - Traditional Chinese Medicine" by the National Higher Education Consultative Committee for Traditional Chinese Medicine Education under the Ministry of Education (http://www.moe.gov.cn/srcsite/A08/moe_740/s3864/201301/t20130105_147172.html), as well as the "Guidelines for the Eight-Year Undergraduate Talent Cultivation in Traditional Chinese Medicine" issued by CDUTCM (Not available online. This document is filed in Office of Educational Administration, and we can provide it if editor request), so we did not register it.

24) Where the full trial protocol can be accessed, if available

Does your paper address CONSORT subitem 24? *

Cite a Multimedia Appendix, other reference, or copy and paste relevant sections from the manuscript (include quotes in quotation marks "like this" to indicate direct quotes from your manuscript), or elaborate on this item by providing additional information not in the ms, or briefly explain why the item is not applicable/relevant for your study

Yes. The full trial protocol is available upon reasonable request.

25) Sources of funding and other support (such as supply of drugs), role of funders

Does your paper address CONSORT subitem 25? *

Copy and paste relevant sections from the manuscript (include quotes in quotation marks "like this" to indicate direct quotes from your manuscript), or elaborate on this item by providing additional information not in the ms, or briefly explain why the item is not applicable/relevant for your study

Yes. "This study was funded by grants JGYB2019008 (Dr. Yang) from the Teaching Reform Project of Chengdu University of Traditional Chinese Medicine; CDUTCM [2020] No. 140 (Dr. Li) from Promotion Plan for Young Teachers of Chengdu University of Traditional Chinese Medicine; S202210633021 (Dr. Xiao) from the Innovation Training Project of Chengdu University of Traditional Chinese Medicine; and ky-2022004 (Dr. Xiao) from the Scientific, Practice, and Innovation Project of Chengdu University of Traditional Chinese Medicine."

X27) Conflicts of Interest (not a CONSORT item)

X27-i) State the relation of the study team towards the system being evaluated

In addition to the usual declaration of interests (financial or otherwise), also state the relation of the study team towards the system being evaluated, i.e., state if the authors/evaluators are distinct from or identical with the developers/sponsors of the intervention.

subitem not at all important

1 ○

2 ○

3 ○

4 ○

5 ◉

essential

清除所选内容

Does your paper address subitem X27-i?

Copy and paste relevant sections from the manuscript (include quotes in quotation marks "like this" to indicate direct quotes from your manuscript), or elaborate on this item by providing additional information not in the ms, or briefly explain why the item is not applicable/relevant for your study

您的回答

About the CONSORT EHEALTH checklist

As a result of using this checklist, did you make changes in your manuscript? *

- (selected) yes, major changes
- yes, minor changes
- no

What were the most important changes you made as a result of using this checklist?

您的回答

How much time did you spend on going through the checklist INCLUDING making changes in your manuscript *

We have spent almost 8 hours to going through the checklist including making changes in the manuscript.

As a result of using this checklist, do you think your manuscript has improved? *

- [x] yes
- [ ] no
- [ ] 其他:

Would you like to become involved in the CONSORT EHEALTH group?

This would involve for example becoming involved in participating in a workshop and writing an "Explanation and Elaboration" document

- [x] yes
- [ ] no
- [ ] 其他:

清除所选内容

Any other comments or questions on CONSORT EHEALTH

您的回答

STOP - Save this form as PDF before you click submit

To generate a record that you filled in this form, we recommend to generate a PDF of this page (on a Mac, simply select "print" and then select "print as PDF") before you submit it.

When you submit your (revised) paper to JMIR, please upload the PDF as supplementary file.

Don't worry if some text in the textboxes is cut off, as we still have the complete information in our database. Thank you!

Final step: Click submit !

Click submit so we have your answers in our database!

提交 清除表单内容

切勿通过 Google 表单提交密码。

此内容不是由 Google 所创建，Google 不对其作任何担保。 举报滥用行为 - 服务条款 - 隐私权政策

Google 表单
